# Octopus-inspired engineered bacteria with a plug-and-play surface display system achieves enhanced tumor-specific colonization and antitumor immunity

**DOI:** 10.1016/j.mmr.2026.100030

**Published:** 2026-04-27

**Authors:** Le-Yang Wu, Jia-Hui Qiu, Xin-Yue Qiao, Lin Li, Li-Yuan Qiao, Chen-Yang Li, Ying Sun, Shu-Hui Zhang, Zeng-Zheng Du, Xiao-Yao Chang, Cheng Cheng, Bo-Hao Wang, Yi-Han Xiao, Lin Lin, Zi-Chun Hua

**Affiliations:** a State Key Laboratory of Pharmaceutical Biotechnology, School of Life Sciences, Nanjing University, Nanjing 210023, China; b Changzhou High-Tech Research Institute of Nanjing University and Jiangsu TargetPharma Laboratories, Inc., Changzhou 213164, Jiangsu, China; c School of Pharmaceutical Sciences, Nanjing Tech University, Nanjing 211816, China; d School of Food and Biological Engineering, Jiangsu University, Nanjing 212013, China; e Faculty of Pharmaceutical Sciences, Xinxiang Medical University, Xinxiang 453002, Henan, China

**Keywords:** Microbial therapeutics, Chemical biological modification, Quorum-sensing (QS), Tumor-targeted delivery, Immune activation

## Abstract

**Background:**

Bacteria-mediated cancer therapy leverages bacteria to modulate the tumor immune microenvironment and deliver therapeutics. However, its clinical application is limited by toxicity, off-target effects, and uncontrolled drug release. Improving tumor targeting and precise payload delivery through rational bacterial engineering is essential for increasing efficacy and safety.

**Methods:**

An attenuated *Salmonella* Δ*htrA*::*luxI*-VNP20009 strain expressing OmpA-SpyTag (AISI-ST) was constructed for the modular surface conjugation of SpyCatcherΔ (SC)-fused quadruple arginine-glycine-aspartic acid (RGD) peptides (named AISI-ST/SC-RGD×4) and for building biointerfaces for enhanced tumor adhesion via RGD-mediated integrin αvβ3 interactions. The tumor-bearing mice received intravenous injections of AISI-ST/SC-RGD×4, and their biodistribution was analyzed using bioluminescence imaging and colony-forming unit (CFU) counts. Quorum-sensing (QS)-regulated high-temperature requirement A (HtrA) and anti-programmed cell death protein 1 (anti-PD1) nanobody expression based on the *LuxI* promoter in strains was validated by Western blotting. Immune responses were assessed using flow cytometry.

**Results:**

The incubation of the fused proteins with the AISI-ST strain for 1 h was sufficient to form a stable biological interface. The quadruple RGD-modified bacteria (AISI-ST/SC-RGD×4) exhibited greater enrichment in various solid tumors and lung metastases with reduced off-target accumulation. QS induced the expression of the HtrA protein within tumors, resulting in enhanced extracellular polysaccharide-mediated immunogenicity to activate immune cells. Further expression of anti-PD1 nanobodies synergistically enhanced antitumor immunity, increasing the percentage of M1 macrophages (MACS) and CD8^+^ T cell proliferation while suppressing M2 MACS and regulatory T cells (Tregs). This approach achieves potent tumor suppression via targeted immune remodeling.

**Conclusions:**

This study presents octopus-inspired engineered bacteria with a “plug-and-display” system and tumor-specific drug delivery that achieves enhanced tumor targeting and potent antitumor effects. This study describes a promising strategy for the precise and safe clinical translation of bacteria-mediated cancer immunotherapy.

## Background

Bacteria-mediated anticancer therapies, a concept with origins dating back more than 4000 years [1], are revolutionizing cancer immunotherapy. These bacteria can proliferate within tumors and effectively reshape the tumor immune microenvironment by stimulating bacterial immunogenic substances [2], [3], [4], [5]. Moreover, genetically engineered bacteria can serve as multifunctional drug delivery vectors capable of delivering antitumor cytokines [6], [7], nucleic acid-based drugs [3], [8], or antibody drugs [9], [10], [11]. Despite the promising potential of bacteria-mediated tumor immunotherapy, several challenges hinder its clinical application. The inherent replication ability and immunostimulatory properties of bacteria pose significant risks of toxicity. When bacteria migrate to normal organs, they can trigger side effects, thereby limiting the maximum permissible dose and potentially preventing the desired therapeutic effects within a safe therapeutic window. This issue has contributed to the cessation of several clinical trials [12], [13]. For example, clinical trials involving patients with metastatic melanoma have shown that attenuated *Salmonella typhimurium* VNP20009 lacks a sufficient targeting capability and fails to induce tumor regression [13], [14]. Additionally, the off-target effects of bacteria, uncontrollable pathogenic stimuli, and premature or unintended drug release can damage healthy tissues [2], [3], [15]. Therefore, improving the tumor-targeting efficiency of bacteria and ensuring precise cargo release are critical. An urgent need exists for the rational, adaptive design of bacterial strains to maximize anticancer efficacy while enhancing targeting precision and safety.

One promising strategy to enhance bacterial tumor targeting involves the display of specific peptides, such as type 1 fimbriae D-mannose-specific adhesin (FimH) [16], CD20-targeting antibodies [17], and cadherin-17 nanobodies [18], on the bacterial outer membrane. Advanced synthetic modification techniques enable this display through bacterial outer membrane proteins (OMPs) [19], [20], specific transmembrane signaling peptides [16], [21], or chemically clicked modifications [10], [22], [23]. Research, including our own, has shown that displaying the arginine-glycine-aspartic acid (RGD) peptide on bacterial or cellular surfaces significantly increases intratumoral enrichment by leveraging the affinity of the peptide for integrin αv and its heterodimers on tumor cells [6], [20], [24]. However, challenges such as protein misfolding, spatial blocking that reduces functionality, and low expression levels are common with OMP or signal peptide fusion expression, necessitating optimization for each specific case [25]. Furthermore, methods such as click chemistry or affinity-based biotin binding, which use organic solvents and other chemical agents, can irreversibly damage bacteria [26]. Recent advancements have led to the development of genetically encoded click chemistry strategies that utilize transmembrane anchors and soluble ligands for separate expression and reassembly, offering a safer and more versatile method for surface display. A notable example is the SpyTag (ST)-SpyCatcher system, which enables straightforward *in vitro* self-assembly of functionalized protein molecules through a simple incubation, resulting in the spontaneous formation of covalent lysine/aspartic acid (Lys/Asp) isopeptide bonds [27], [28], [29]. To increase covalent conjugation precision, SpyCatcherΔ (SC) was created, which lacks 21 residues at the N-terminus and 14 residues at the C-terminus compared with the original SpyCatcher [30].

Inspired by the robust and stable adhesion properties of octopus suction cups, this study aimed to develop a multifunctional bacterial therapeutic platform by integrating genetic engineering, covalent surface modification, and synthetic biology strategies, with the goals of enhancing tumor-targeting capability, enabling controllable intratumoral activation, and improving the precision of bacteria-mediated tumor immunotherapy. To achieve this, we designed an integrated system that combines a ST-SC covalent conjugation module, a quorum-sensing (QS)-responsive bacterial activation circuit, and a localized immune checkpoint blockade release strategy. Specifically, an engineered attenuated *Salmonella ΔhtrA::luxI*-VNP20009 strain (AISI), was modified to display ST protein on its outer membrane as an anchoring motif, enabling the covalent attachment of a fusion protein containing dimerized RGD peptides and SC protein to enhance tumor-specific adhesion. In addition, a QS-based regulatory circuit was introduced to promote bacterial activation within the tumor microenvironment, while therapeutic proteins, such as an anti-PD-1 nanobody, were designed for localized release at tumor sites, thereby maximizing antitumor efficacy while minimizing systemic toxicity.

## Methods

### SpyTag-SpyCatcherΔ binding assay

To evaluate ST-SC binding, AISI-ST bacteria (OD600=1.0) were incubated with SC-enhanced green fluorescent protein (eGFP) for 2 h. After washing with phosphate-buffered saline (PBS), binding was assessed using multiple approaches. GFP fluorescence intensity was measured with a microplate reader (Biotek, Winooski, USA) at Ex/Em 488/525 nm. Flow cytometry (BD FACSCanto II, USA) quantified surface GFP fluorescence. For imaging, AISI-RFP-ST [constitutively expressing red fluorescent protein (RFP)] was co‑incubated with SC‑eGFP, washed, and stained with Hoechst (KGA1804-10, KeyGEN BioTECH, Nanjing, China). Samples were imaged via fluorescence microscope (Zeiss, Germany) in fluorescein isothiocyanate (FITC) and 4’,6-diamidino-2-phenylindole (DAPI) channels to visualize SC‑eGFP binding.

### Adhesion assay of engineered bacteria

For competitive inhibition assays using exogenous RGD peptides, tumor cells were preincubated with varying concentrations of RGD peptide (HY-P0278, MCE, Shanghai, China) for 30 min, followed by bacterial coincubation and adhesion quantification as described above. To prepare the surfaces for the strain adhesion assays, the recombinant mouse integrin αvβ3 (TGAV & ITGB3) heterodimer protein (HY-P700761, MCE, USA) was first diluted to 10 μg/ml in 50 mmol/L carbonate buffer (pH 9.6). Subsequently, 96-well plates were coated with this protein solution at a volume of 100 μl per well and incubated overnight at 4 °C. After washing with PBS with Tween-20 (PBST), 3% bovine serum albumin (BSA) was added to each well, and the plate was closed for 3 h at 37 °C. After washing, 100 μl of different strains diluted in a serum gradient [engineered strains expressing RFP were used at a final concentration of 10^6^ colony-forming unit (CFU)/100 μl] was added to the wells. Following a 30-min incubation and subsequent washing of the wells, the adhered bacteria were visualized via fluorescence microscopy.

### Extracellular polysaccharide assays

To quantify the extracellular polysaccharide (EPS) produced by bacterial strains, single colonies were picked from agar plates and incubated overnight at 37 °C. Equal volumes of bacterial cultures (OD600=1.0) were centrifuged, and the pellets were resuspended in EPS extraction reagent (EX1750, Solarbio, China). The resuspension was incubated in an 80 °C water bath for 6 h. After centrifugation at 10,000×*g*, the supernatant containing the extracted EPSs was collected. The EPS concentration was determined via a phenol-sulfuric acid colorimetric assay performed with a polysaccharide quantification kit (ZC-S0885, ZCIBIO, Shanghai, China). The absorbance at 490 nm (OD490) was measured for each sample, and the polysaccharide content was calculated on the basis of a standard curve.

### Animal experiments

BALB/c (female, 4–5 weeks old, *n*=145) and C57BL/6 (female, 6–8 weeks old, *n*=175) mice were purchased from Cavens Laboratory Animal Co., Ltd. (Changzhou, China). Female mice were used because they are not stimulated by androgens, which prevents experimental interference caused by factors such as estrus, and the corresponding results are more widely accepted by other researchers [31]. Animals were randomly assigned to experimental or control groups, and investigators were blinded to group allocation during the experiments. All procedures were conducted according to ethical guidelines and were approved by the Institutional Animal Care and Use Committee (IACUC) of Nanjing University (IACUC-2003167).

### Real-time quantitative polymerase chain reaction assays

The total RNA was extracted via a total RNA isolation kit (RC112, Vazyme, Nanjing, China) and then reverse transcribed into cDNA via a reverse transcription kit (M16315, Thermo Scientific, USA). For quantitative PCR, the cDNA was mixed with a qPCR mixture (Q111-02, Vazyme, Nanjing, China) and specific primers (sequences are provided in Additional file 1**:** Table S1). Real-time quantitative polymerase chain reaction (RT-qPCR) was performed in a StepOnePlus Real-Time PCR system (Applied Biosystems, USA), and AceQ qPCR SYBR Green Master Mix (Q221-01, Vazyme, Nanjing, China) was used for gene amplification. For the detection of reactive oxygen species (ROS) and nitric oxide (NO), the ROS probe 2’,7’-dichlorodihydrofluorescein diacetate (H2DCFDA, HY-D0940, MCE, USA) and NO probe 3-amino,4-aminomethyl-2’,7’-difluorescein, diacetate (DAF-FM DA, S0019S, Biyuntian, Shanghai, China) were used in accordance with the manufacturer’s instructions. A Toll-like receptor 4 (TLR4) inhibitor (S7455, Resatorvid, Selleck, Shanghai, China) was used to block bacterial activation in macrophages (MACS).

### Western blotting assays

Total protein was collected as described previously [9]. The concentrations of total proteins were adjusted to the same concentration. Twenty microliters of the mixture (approximately 15 μg of protein) was used for Western blotting analysis. The following antibodies were used: His tag antibody (SAB1305538, Sigma-Aldrich, USA), FLAG tag antibody (F1804, Sigma-Aldrich, USA), anti-myeloid differentiation primary response 88 (MyD88) antibody (A21905, ABclonal, Wuhan, China), anti-c-Jun N-terminal kinase (JNK) antibody (#9252, CST, Boston, USA), anti-phosphorylated JNK antibody (AP0631, ABclonal, Wuhan, China), anti-inhibitor of κB (IκBα) antibody (ABclonal, A24909, Wuhan, China), anti-phosphorylated IκBα antibody (AP0999, ABclonal, Wuhan, China), anti-extracellular signal-regulated kinase 1 (ERK1) antibody (610030, BD, USA), anti-phosphorylated ERK1/2 antibody (AP0484, Bioworld Tech, Nanjing, China), anti-GAPDH antibody (AC027, ABclonal, Wuhan, China), and anti-tubulin antibody (A12289, ABclonal, Wuhan, China). A phosphatase inhibitor cocktail (20109ES05, Yeasen, China) was used.

### Enzyme-linked immunosorbent assay

Various engineered bacterial strains were injected via the tail vein into B16-F10 mice. After administration, the serum was collected. The tumors were mixed with tissue lysis reagent (abs9225, Absin, Shanghai, China) at a concentration of 10 mg tissue/50 μl and homogenized with the tissue lysis reagent. The supernatant was collected after centrifugation. Tumor necrosis factor-α (TNF-α) and interferon-γ (IFN-γ) in the serum and tumor lysates of different groups of mice were detected via a mouse TNF-α enzyme-linked immunosorbent assay (ELISA) Kit (BY-EM220852, BYabscience, China) and an IFN-γ ELISA Kit (BY-EM220140, BYabscience, China). Other methods are displayed in the Additional file 2**: Methods**.

### Statistical analysis

Data analysis was performed using GraphPad Prism software, version 9.0. Student’s *t*-test was used for comparisons between two groups, and one-way analysis of variance (ANOVA) was used for comparisons involving more than three groups, followed by Dunnett’s multiple comparisons test. The data are expressed as the mean±standard deviation (SD) from a representative experiment out of two or three technical replicates. Statistical significance was determined as follows: ^⁎^*P*<0.05, ^⁎⁎^*P*<0.01, ^⁎⁎⁎^*P*<0.001, and ^⁎⁎⁎⁎^*P*<0.0001. Non-significant results are labeled ns. Schematic images and graphics were created using Adobe Illustrator software.

## Results

### Construction of the ST/SC-based octopus tentacle (ST/SC-RGD×4) and evaluation

**Fig. 1:**
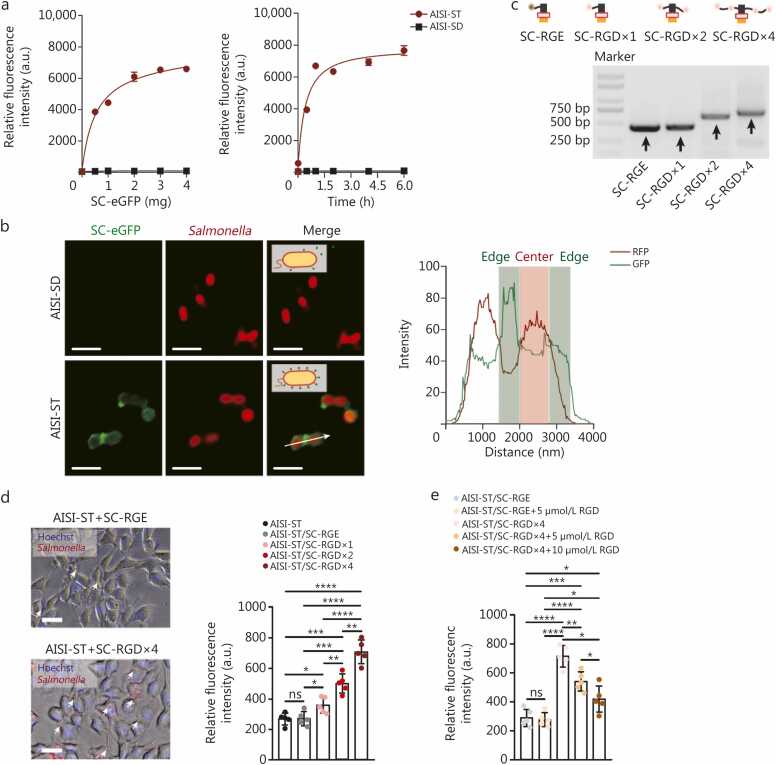
**Screening and evaluation of bacterial outer membrane ST/SC-RGD tentacles**. **a** Changes in the total fluorescence intensity of the strains after the coincubation of different amounts of SC-eGFP (0.5, 1, 2, 3, and 4 mg) with 10^8^ CFU of the AISI-ST or AISI-SD strains for 1 h (left panel). Changes in the total fluorescence intensity of the strains after the coincubation of 3 mg of SC-eGFP with 10^8^ CFU of the AISI-ST or AISI-SD strains for different durations (0.01, 0.5, 1, 2, 4, or 6 h) (right panel). *n*=3. **b** Representative fluorescence images and quantitative analysis of the AISI-ST or AISI-SD strains after the coincubation with SC-eGFP. SC-eGFP bound uniformly and efficiently to the outer membrane of the AISI-ST strain but did not bind to that of the AISI-SD strain. Scale bar=10 µm. White arrow indicates the direction and region of analysis. **c** Agarose gel electrophoresis of different plasmids with SC, SC-RGD×1, SC-RGD×2, and SC-RGD×4 sequences. **d** Different engineered strains, including AISI-ST, AISI-ST/SC-RGE, AISI-ST/SC-RGD×1, AISI-ST/SC-RGD×2, and AISI-ST/SC-RGD×4, were coincubated with B16-F10 tumor cells at an multiplicity of infection (MOI) of 100. Free bacteria were removed, and the total fluorescence intensity of the strains adhering to tumor cells was detected (left panel). A chassis-engineered strain that stably expresses RFP was used. Representative fluorescence images of the AISI-ST/SC-RGE and AISI-ST/SC-RGD×4 strains bound to tumor cells are shown (right panel). Hoechst was used to stain the cell nuclei. The white arrows indicate engineered strains adhering to tumor cells. Scale bar=40 µm. **e** Efficacy of the adhesion of the AISI-ST/SC-RGE strain and the AISI-ST/SC-RGD×4 strain to B16-F10 tumor cells was assessed after prespiking the free RGD peptide (5 or 10 µmol/L) in the medium. *n*=5. The data are presented as the mean±SD. ^⁎^*P*<0.05, ^⁎⁎^*P*<0.01, ^⁎⁎⁎⁎^*P*<0.0001, ns non-significant. a.u. Arbitrary units; AISI. Attenuated *Salmonella* Δ*htrA*::*luxI*-VNP20009 strains; AISI-SD. AISI-ST DA; OmpA. Outer membrane proteins A; ST. SpyTag; SC. SpyCatcherΔ; DA. SpyTag Asp117 Ala mutant; RGD. Arginine-glycine-aspartic acid; RGE. Arginine-glycine-glutamic acid; eGFP. Enhanced green fluorescent protein; RFP. Red fluorescent protein.

The fusion of target proteins with bacterial OMPs, such as outer membrane protein A (OmpA), facilitates their presentation on the bacterial surface [20]. We hypothesized that the ST protein could be anchored to the bacterial outer membrane through a similar strategy, allowing it to be spliced to SpyCatcherΔ-modified target proteins (abbreviated as SC) through a simple coincubation. Based on this conjugation, multiple RGD motifs could be further covalently displayed on the bacterial surface to enhance the specific recognition of tumor cells by the bacteria (Additional file 1**:** Fig. S1). We engineered AISI strains, which are attenuated *Salmonella* chassis strains that exhibit excellent safety profiles [32]. We deleted the *ompA* gene from the genome of the AISI strain and introduced a plasmid for the continuous expression of OmpA-ST protein, creating the AISI-ST-engineered strain (abbreviated as AISI-ST; Additional file 1**:** Fig. S1). The ST was inserted into the third outwardly protruding loop of OmpA in the AISI strain. The AISI-SD strain, which contains the inserted ST DA structure (abbreviated as SD, a ST mutant that does not bind to the SpyCatcher) [27], serves as a control (Additional file 1**:** Fig. S2a). This engineered modification did not significantly alter the strain morphology or growth rate (Additional file 1**:** Fig. S2b**, c**). The SC-eGFP fusion protein can be generated by constructing and expressing an SC-eGFP fusion gene (Additional file 1**:** Fig. S2d). We then evaluated the clicking efficiency of the SC-eGFP in the AISI-ST strain. The SC-eGFP protein effectively spliced with the AISI-ST strain in a dose- and time-dependent manner, yielding the AISI-ST/SC-eGFP strain (Fig. 1**a**). We verified that incubating 3 mg of SC-eGFP with ^8^ CFU of the strain for 1 h resulted in a uniform and saturated distribution of the engineered protein on the bacterial outer membrane (Fig. 1**a, b**). The number of SC-eGFP molecules per bacterium was determined using a standard curve correlating fluorescence intensity with protein mass, together with a formula based on molar quantity (detailed procedures are provided in the Additional file 2**: Methods**). Each bacterium displayed approximately 4.706×10^8^ eGFP molecules (about 3.428×10^-5 ^ng protein per bacterium) on its surface (Fig. 1**b**; Additional file 1**:** Fig. S2e). Recombinant proteins can be uniformly fused to the surface of bacteria, ensuring sufficient contact between the protein and the receptor of the target cell (Fig. 1**b**). Moreover, the AISI-ST/SC-eGFP strain stably maintained the SC-eGFP protein in serum for more than 80 min (Additional file 1**:** Fig. S2f), which is well beyond the duration of a typical *in vivo* circulation cycle [33], increasing the potential for effective receptor targeting by OMPs.

We aimed to express the RGD peptide fused with SC and splice it onto the bacterial outer surface. Previous constructs of multimeric RGDs, particularly quadruple RGDs, displayed superior *in vivo* tumor-targeting properties but were synthesized primarily chemically [34]. Thus, we developed a biologically expressible quadruple RGD-SC fusion protein (SC-RGD×4) to achieve a similar effect (Additional file 1**:** Fig. S3a). Lesser oligomer versions, SC-RGD×2 and SC-RGD×1, were also constructed (Fig. 1**c**). The N-terminus and C-terminus of the SC protein were connected to the RGD peptide via a rigid linker, and a flexible linker was used to connect the second RGD peptide extending outward from the RGD peptide to ultimately ensure overall stability (Additional file 1**:** Fig. S3b). These proteins were incubated with 10^8^ CFU of the AISI-ST strains for 1 h to create various protein-modified strains, including AISI-ST/SC-RGD×1, AISI-ST/SC-RGD×2, and AISI-ST/SC-RGD×4. The SC-RGD×4 peptide is capable of covalently binding to the head domain of OmpA-ST on the bacterial surface (Additional file 1**:** Fig. S3c**;** Additional file 3**:** Video). An individual AISI-ST/SC-RGD×4 strain was estimated to display about 1.88×10^9^ RGD peptides on its surface, based on conversion of the measured signal to an equivalent number of eGFP molecules using a reference calibration. These peptides are expected to increase bacterial binding to tumor cells through interactions with αvβ integrins, which are abundant on tumor cell surfaces. We confirmed this hypothesis by testing the adherence of the strains to B16-F10 mouse melanoma cells, which express high levels of αvβ integrin according to reports [35]. The AISI-ST/SC-arginine-glycine-glutamic acid (RGE) strain, which lacks αvβ3 binding activity [36], served as a control. The results indicated that the adhesion properties of the AISI-ST/SC-RGE strain did not differ significantly from those of the unmodified AISI-ST strain (Fig. 1**d**). In contrast, the AISI-ST/SC-RGD×1 strain exhibited a 1.32-fold increase in tumor adhesion, highlighting the role of the RGD modification in enhancing bacterial adhesion to tumor cells. More notably, the adhesion of the AISI-ST/SC-RGD×2 and AISI-ST/SC-RGD×4 strains increased to an even greater extent (1.84-fold and 2.61-fold, respectively) (Fig. 1**d**), suggesting that the increase in adhesion was linked to the increasing number of RGD peptides. This trend was disrupted when tumor cells were pretreated with free RGD peptides, which significantly reduced the degree of adhesion (Fig. 1**e**). The adhesion of different strains to surfaces coated with recombinant αvβ3 integrin was subsequently assessed. The results revealed that the adhesion of the AISI-ST/SC-RGD×4 strain to the recombinant αvβ3 integrin-coated surfaces was 4.30-fold greater than that of the AISI-ST/SC-RGE strain (Additional file 1**:** Fig. S3d**, e)**. This increase was not observed on surfaces coated with BSA alone, further indicating that bacterial adhesion is mediated by RGD-αvβ3 interactions. Compared with the AISI-ST/SC-RGE×4 strain, which possesses the same linker sequences as the AISI-ST/SC-RGD×4 strain, the AISI-ST/SC-RGE×4 strain did not display enhanced tumor cell targeting, suggesting that the linker sequences were not the source of the enhanced tumor specificity (Additional file 1**:** Fig. S3f). Moreover, the adhesion of the AISI-ST/SC-RGD×4 strain consistently improved across various tumor cell lines, including 4T1 mouse breast cancer cells and H22 mouse hepatocellular carcinoma cells, which also express high levels of αvβ integrin (Additional file 1**:** Fig. S3g). These findings confirm the effectiveness of rapidly indirect coupling multiple RGD peptides onto bacterial surfaces to increase bacterial recognition and adhesion to tumor cells.

### The AISI-ST/SC-RGD strain achieves strong targeting and enrichment of multiple tumor types

**Fig. 2:**
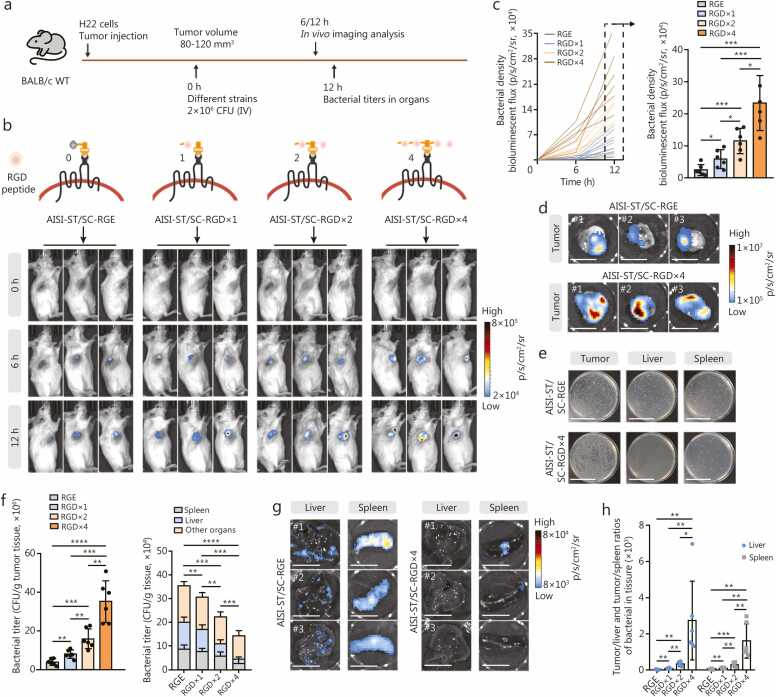
**Enhanced intratumoral enrichment of RGD peptide-modified strains**. **a** Schematic of the analysis of the *in vivo* distribution of different strains, including the AISI-ST/SC-RGE strain, AISI-ST/SC-RGD×1 strain, AISI-ST/SC-RGD×2 strain, and AISI-ST/SC-RGD×4 strain (2×10^6^ CFU per mouse), in a subcutaneous xenograft tumor model. **b** Schematic of multiple RGD “suckers” biospliced onto the outer membrane of AISI-ST strain (top panel). Representative *in vivo* imaging images of several RGD-modified strains at 6 and 12 h after administration to tumor-bearing mice are shown (bottom panel). The strains were engineered to express the spontaneous bioluminescent protein LuxCDABE for *in vivo* tracing. **c** Curve showing the bioluminescence flux of different strains in the tumors of each mouse after different treatments (left panel) and a comparison of the bioluminescence intensity in different groups of mice 12 h after treatment (right panel). *n*=6. **d** Representative images of tumors 12 h after the administration of the AISI-ST/SC-RGE or AISI-ST/SC-RGD×4 strains to H22 cell subcutaneous xenograft model mice. Scale bar=1 cm. The labels #1, #2, and #3 in the images denote different representative individual mice. **e** Representative photographs of bacterial growth on plates after dilution of samples collected from the tumors, spleens, and livers of the AISI-ST/SC-RGE or AISI-ST/SC-RGD×4 groups of mice in (b). Scale bar=5 cm. **f** Comparison of bacterial titers at 12 h after administration in the tumor tissues (left panel) and the liver, spleen, and other normal organs (including the heart, lung, and kidney) (right panel). *n*=6. **g** Representative images of the liver and spleen 12 h after the administration of the AISI-ST/SC-RGE or AISI-ST/SC-RGD×4 strains to H22 cell subcutaneous xenograft model mice. Scale bar (left)=2 cm, Scale bar (right)=1 cm. The labels #1, #2 and #3 in the images denote different representative individual mice. **h** The ratio of bacterial colony-forming unit (CFU) in tumors to those in the liver and spleen per gram of tissue at 12 h. The mean tumor/liver and tumor/spleen ratios are shown. The data are reported as the mean±SD. ^⁎^*P*<0.05, ^⁎⁎^*P*<0.01, ^⁎⁎⁎^*P*<0.001, ^⁎⁎⁎⁎^*P*<0.0001, ns non-significant. IV. Intravenously; AISI. Attenuated *Salmonella* Δ*htrA*::*luxI*-VNP20009 strains; ST. SpyTag; SC. SpyCatcherΔ; RGD. Arginine-glycine-aspartic acid; RGE. Arginine-glycine-glutamic acid; WT. Wild type.

Next, AISI-ST/SC-RGD strains displaying multiple RGD peptides were hypothesized to exhibit enhanced tumor recognition and *in vivo* enrichment. We administered four strains, namely, AISI-ST/SC-RGE, AISI-ST/SC-RGD×1, AISI-ST/SC-RGD×2, and AISI-ST/SC-RGD×4, via intravenous injection into mice with H22 cell subcutaneous xenografts (Fig. 2**a**). Each strain was engineered to continuously express a luminescent reporter gene (*LuxCDABE*) for real-time tracking. *In vivo* imaging revealed the presence of detectable bacteria in tumors within 6 h after administration, with the levels increasing over time (Fig. 2**b, c**). The tumor enrichment efficiency was positively correlated with the number of RGD peptides on the bacterial surface. Compared with that of AISI-ST/SC-RGE, the luminescence intensity detected at the tumor site at 12 h after administration increased 2.41-fold for AISI-ST/SC-RGD×1, 4.74-fold for AISI-ST/SC-RGD×2, and 9.64-fold for AISI-ST/SC-RGD×4 (Fig. 2**b, c**). Imaging of tumor tissues showed the superior intratumor enrichment of AISI-ST/SC-RGD×4 strain (Fig. 2**d**). An analysis of the bacterial titer in tumor and normal tissues (including liver and spleen) revealed that multiplex RGD significantly increased bacterial-specific enrichment within tumors (Fig. 2**e**). Compared with the AISI-ST/SC-RGE strain, a significantly increased bacterial titer of the AISI-ST/SC-RGD×4 strain was detected in tumors 3 h after administration, essentially reaching a peak within 24 h (120 h for AISI-ST/SC-RGE strain). A greater tumor bacterial titer of the AISI-ST/SC-RGD×4 strain was presented within 120 h after administration (Additional file 1**:** Fig. S4a). Most off-target organs, including the liver and spleen, presented detectable off-target bacterial presence after the administration of the AISI-ST/SC-RGE strains. Notably, the RGD modification markedly reduced this off-target effect, with AISI-ST/SC-RGD×4 resulting in significantly lower titers in these organs, only 40.8% of those observed with the AISI-ST/SC-RGE strain (Fig. 2**f**). Following administration of the AISI-ST/SC-RGE strain, bacterial titers in the liver and spleen reached 11.0×10^4^ CFU/g and 8.9×10⁴ CFU/g, respectively. In contrast, titers in the AISI-ST/SC-RGD×4 group were reduced to 1.7×10⁴ CFU/g (an 84.5% reduction) in the liver and 2.8×10⁴ CFU/g (a 68.5% reduction) in the spleen (Fig. 2**f, g**). Despite the off-target effects of the strains, due to the naturally low immunogenicity of the chassis strain AISI [32], both the AISI-ST/SC-RGE strain and the AISI-ST/SC-RGD×4 strain displayed excellent biocompatibility (Additional file 1**:** Fig. S4b). The tumor-targeting efficiency, defined as the ratio of the bacterial titer within the tumor to that within normal organs (e.g., liver or spleen), was significantly greater for the AISI-ST/SC-RGD×4 strain, with a 96.80-fold greater efficiency for the tumor/liver and 34.60-fold greater efficiency for the tumor/spleen than the AISI-ST/SC-RGE strain (Fig. 2**h**). This enhanced targeting was attributed to increased bacterial enrichment within tumors combined with a reduced off-target distribution.

**Fig. 3:**
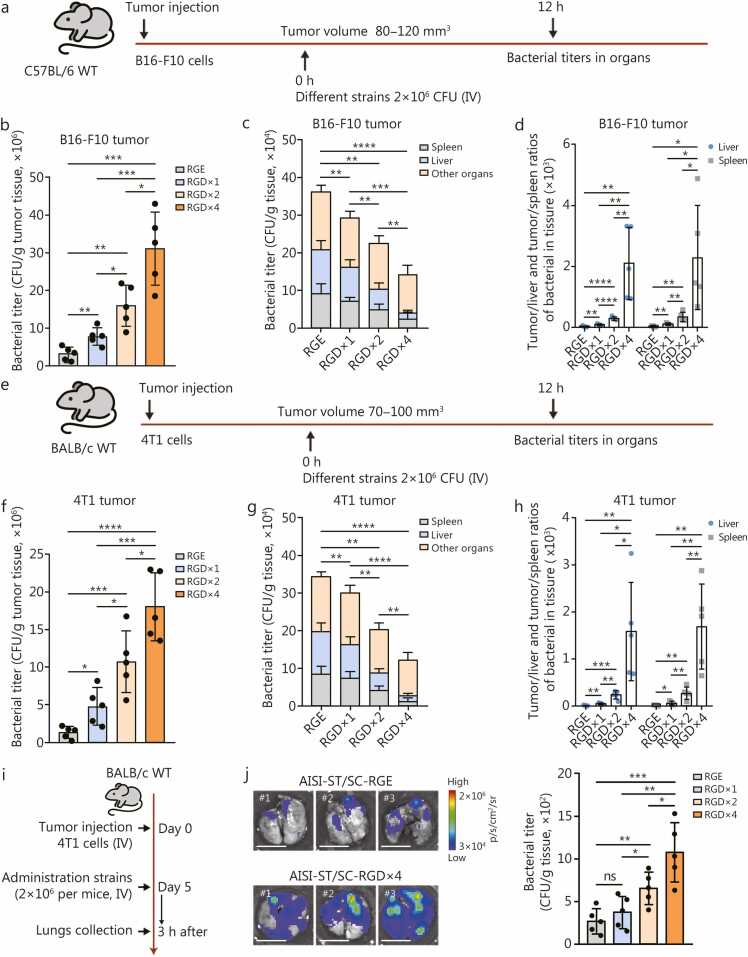
**The AISI-ST/SC-RGD×4 strain has an excellent tumor-targeting ability in multiple tumor models**. **a** Schematic of the *in vivo* distribution analysis of different strains, including AISI-ST/SC-RGE, AISI-ST/SC-RGD×1, AISI-ST/SC-RGD×2, and AISI-ST/SC-RGD×4 (2×10⁶ CFU per mouse, IV), in an in situ melanoma mouse tumor model (**a-d**). **b** Comparison of bacterial titers at hour 12 in melanoma tumor tissue. *n*=5. **c** Comparison of bacterial titers at hour 12 in liver, spleen, and other normal organs (including heart, lung, and kidney). *n*=5. **d** The ratio of bacterial CFU in tumors to liver and spleen per gram of tissue at hour 12. The mean values of the tumor/spleen (left) and tumor/liver (right) ratios are shown. **e** Schematic of the *in vivo* distribution analysis of different strains, including AISI-ST/SC-RGE, AISI-ST/SC-RGD×1, AISI-ST/SC-RGD×2, and AISI-ST/SC-RGD×4 (2×10⁶ CFU per mouse, IV), in an in situ breast cancer (4T1) tumor model (**e-h**). **f** Comparison of bacterial titers at hour 12 in breast tumor tissue. *n*=5. **g** Comparison of bacterial titers at hour 12 in liver, spleen, and other normal organs (including heart, lung, and kidney). *n*=5. **h** The ratio of bacterial CFU in tumors to liver and spleen per gram of tissue at hour 12. The mean values of the tumor/spleen (left) and tumor/liver (right) ratios are shown. **i** Schematic of the analysis of the *in vivo* distribution of different strains, including the AISI-ST/SC-RGE strain, AISI-ST/SC-RGD×1 strain, AISI-ST/SC-RGD×2 strain, and AISI-ST/SC-RGD×4 strain (2×10^6^ CFU per mouse, IV, intravenously), in a 4T1 breast cancer lung metastasis mouse model. **j** Representative images of the lungs of 4T1 cell lung metastasis model mice 3 h after the administration of different strains expressing LuxCDABE (left panel). A histogram comparing the number of bacteria in the lungs of mice from different groups is shown (right panel). *n*=5. Scale bar=1 cm. The labels #1, #2 and #3 in the images denote different representative individual mice. Data are presented as mean±SD. ^⁎^*P*<0.05, ^⁎⁎^*P*<0.01, ^⁎⁎⁎^*P*<0.001, ^⁎⁎⁎⁎^*P*<0.0001. CFU. Colony-forming unit; AISI. Attenuated *Salmonella* Δ*htrA*::*luxI*-VNP20009 strain; ST. SpyTag; SC. SpyCatcherΔ; RGD. Arginine-glycine-aspartic acid; RGE. Arginine-glycine-glutamic acid; IV. Intravenous; WT. Wild type.

We further validated the tumor-targeting properties of the engineered bacterium in *in situ* melanoma (B16-F10) and breast cancer (4T1) mouse models (Fig. 3**a, e**). The results revealed significantly enhanced tumor enrichment for the RGD peptide-modified bacteria. Compared with the AISI-ST/SC-RGE strain, the AISI-ST/SC-RGD×1 strain exhibited a 2.48-fold increase in the melanoma titer and a 1.52-fold increase in the breast cancer tumor titer. Similarly, the AISI-ST/SC-RGD×2 strain presented 5.07-fold (melanoma) and 3.41-fold (breast cancer) increases, whereas the AISI-ST/SC-RGD×4 strain presented 9.87-fold (melanoma) and 5.73-fold (breast cancer) increases (Fig. 3**b, f**). The enrichment of the RGD-modified strains was also lower in normal organs (Fig. 3**c, g**), with the most significant increase in tumor targeting observed for the AISI-ST/SC-RGD×4 strain: 65.70-fold (tumor/spleen) and 74.90-fold (tumor/liver) in the melanoma models and 112.50-fold (tumor/spleen) and 132.80-fold (tumor/liver) in the mammary cancer models (Fig. 3**d, h**). Furthermore, the targeting efficacy of these engineered strains toward lung metastatic tumors was evaluated (Fig. 3**i**). Compared with that of the AISI-ST/SC-RGE strain, the enrichment of the AISI-ST/SC-RGD×4 strain in lung metastases was also significantly greater (Fig. 3**j**). These findings underscore the superior *in vivo* tumor enrichment ability of the AISI-ST/SC-RGD×4 strain and its ability to minimize off-target effects on healthy tissues. Subsequent studies will focus on the AISI-ST/SC-RGD×4 strain.

### The AISI-ST/SC-RGD×4 strain was combined with a dynamic virulence regulator for efficient and precise anticancer treatment

Compared with the AISI-ST strain, the intravenously injected AISI-ST/SC-RGD×4 strain capitalized on the tumor-specific recognition and adhesion properties of the RGD peptide, achieving earlier and more precise intratumor enrichment (Fig. 2). When the AISI chassis bacteria were utilized, the biosafety profile of the AISI-ST/SC-RGD×4 strain was excellent (Additional file 1**:** Fig. S5a). However, high intratumor titers of the AISI-ST/SC-RGD×4 strain did not correspond to increased anticancer effects (Additional file 1**:** Fig. S5b). We hypothesized that re-expressing HtrA proteins via a QS system could increase the anticancer efficacy of the AISI-ST/SC-RGD×4 strain (Additional file 1**:** Fig. S6a), similar to how an octopus changes color in response to specific stimuli [11]. We engineered the AISI-pLuxI-LuxCDABE-ST strain (abbreviated as AISI-L-ST), a population-sensing-mediated bioluminescence-producing (based on *LuxCDABE* gene cluster) strain, to test this hypothesis. QS-induced expression systems have attracted increasing attention owing to their self-inducibility and rapid signal amplification upon activation. The AISI strain benefits from the genomic integration of the *LuxI* gene, which encodes the key enzyme responsible for producing the QS autoinducer, thereby conferring enhanced QS sensitivity. Expression is typically initiated when bacterial density reaches approximately 10⁷ CFU/ml or when local autoinducer concentrations exceed a threshold level [32], [37]. The anti-loss element Axe/Txe was inserted into the plasmids to ensure a durable engineered phenotype of the strain [38]. *In vivo* imaging revealed that compared with the AISI-L-ST/SC-RGE strain, the AISI-L-ST/SC-RGD×4 strain initiated bioluminescence earlier **(**Additional file 1**:** Fig. S6b**, c**). At 36 h after administration, the intratumoral bioluminescence intensity of the AISI-L-ST/SC-RGD×4 strain was 27.8 times higher than that of the AISI-L-ST strain (Additional file 1**:** Fig. S6b**, c**).

**Fig. 4:**
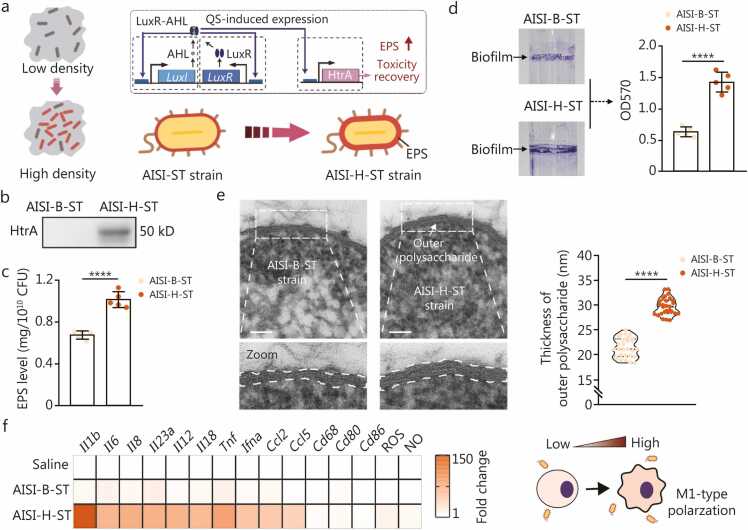
**HtrA re-expression by the AISI-H-ST strain achieves efficient anticancer therapy**. **a** Schematic of the controllable extracellular polysaccharide (EPS)-mediated virulence recovery circuit based on AISI-ST chassis bacteria. The expression of the *HtrA* gene is controlled by the *LuxI* promoter, enabling bacterial HtrA protein re-expression only at high population densities. This novel strain is referred to as AISI-H-ST. **b** Immunoblot analysis of HtrA expression in the AISI-B-ST and AISI-H-ST strains *in vitro* at high bacterial densities. **c** Comparison of EPS levels in the AISI-B-ST and AISI-H-ST strains at the same bacterial count. *n*=6. **d** Representative images of test tubes stained for biofilms of the AISI-B-ST and AISI-H-ST strains and quantitative comparisons after biofilm elution. *n*=5. **e** Representative transmission electron microscopy images of ruthenium red-stained strains showing EPS on the outer membrane (left panel). The AISI-B-ST strain served as a blank control and was transfected with an empty plasmid. HtrA re-expression results in a rescued EPS structure on the cell surface of the AISI-H-ST strain. The white dashed box and white arrows indicate the outer membrane of the bacteria. Scale bar=100 nm. The violin plot shows a shift in the outer membrane thickness between the two strains (right panel). **f** M1-type macrophage-related gene expression and key effector molecules (including ROS and NO) were detected after the different strain were cocultured with M0-type macrophages for 6 h. Compared with the AISI-B-ST strain, the AISI-H-ST strain induced more pronounced M1-type polarization of the macrophages. The data are presented as the mean±SDs. ^⁎^*P*<0.05, ^⁎⁎^*P*<0.01, ^⁎⁎⁎^*P*<0.001, ^⁎⁎⁎⁎^*P*<0.0001, ns non-significant. QS. Quorum-sensing; AHL. N-acyl homoserine lactone; CFU. Colony-forming unit; HtrA. High-temperature requirement A; AISI. Attenuated *Salmonella* Δ*htrA*::*luxI*-VNP20009 strain; ST. SpyTag; SC. SpyCatcherΔ; RGD. Arginine-glycine-aspartic acid; RGE. Arginine-glycine-glutamic acid; B. Blank; H. HtrA; Tnf. Tumor necrosis factor; ROS. Reactive oxygen species; NO. Nitric oxide; WT. Wild type; IV. Intravenous.

**Fig. 5:**
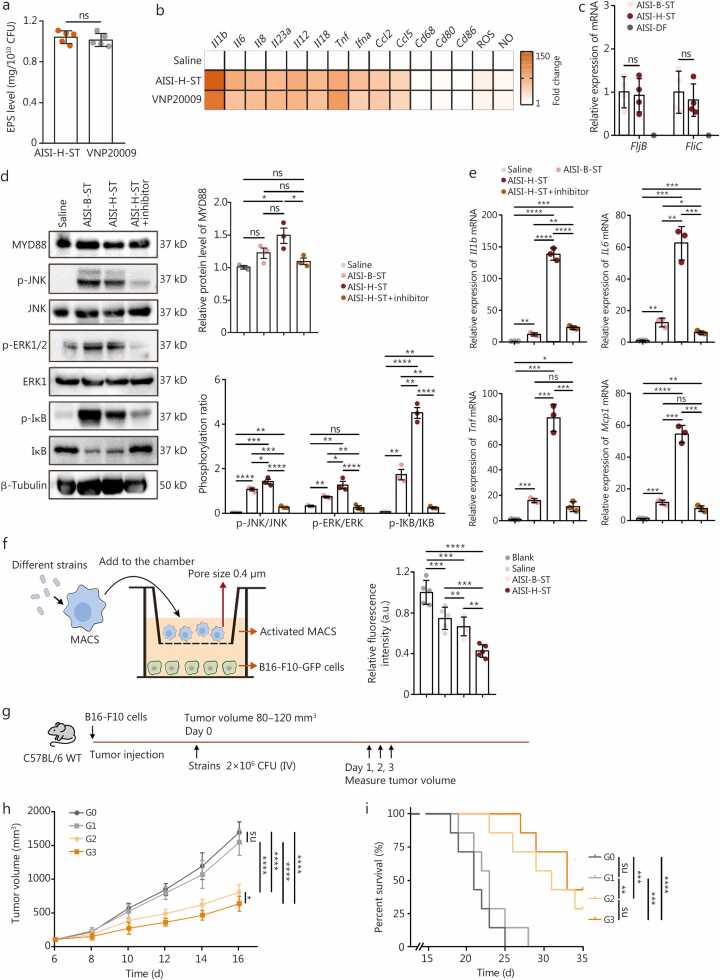
**The AISI-H-ST strain induces macrophage activation through EPS-mediated activation of the TLR4 signaling pathway**. **a** Comparison of EPS levels in the AISI-H-ST and VNP20009 strains at the same bacterial count. *n*=6. **b** Detection of M1-type macrophage-related gene expression and key effector molecules (including ROS and NO) after co-culturing AISI-H-ST and VNP20009 strains with M0-type macrophages for 6 h. **c** The expression of representative flagella-associated genes, including *fljB* (flagellar phase 2 gene B) and *fliC* (flagellar phase 2 gene C)*,* was examined in the AISI-B-ST and AISI-H-ST strains. A Δ*fljB*&Δ*fliC* mutant (designated AISI-DF), which lacks functional flagella, served as the negative control. **d** The activation status of ERK/JNK and NF-κB signaling pathways in macrophages stimulated with strains for 6 h. The representative Western blot analysis and corresponding quantitative analysis of protein expression in macrophages after 6 h of stimulation with the indicated strains. **e** Detection of represented M1-type macrophage-related gene expression, including *Il1b*, *Il6*, *Tnf,* and *Mcp1*, after co-culturing different strains with M0-type macrophages for 6 h. A small-molecule inhibitor of Toll-like receptor 4 (TLR4) signaling (Resatorvid) was used. TLR4 blockade attenuates the activation effect of AISI-H-ST strain on macrophages. **f** Schematic of co-culturing macrophages activated by different strains with B16-F10-GFP tumor cells (left). After co-culturing for 12 h, the differences in relative fluorescence intensity of tumor cell among the different groups were compared (right). Macrophages activated by the AISI-H-ST strain significantly inhibited the growth of tumor cells in the lower chamber. *n*=5. **g** Schematic representation of different strains, including AISI-B-ST/SC-RGD×4 (AISI-B-ST strain spliced with the SC-RGD×4 protein, G1 group), AISI-H-ST/SC-RGE (AISI-H-ST strain spliced with the SC-RGE protein, G2 group), and AISI-H-ST/SC-RGD×4 (AISI-H-ST strain spliced with SC-RGD×4 protein, G3 group), used for antitumor therapy. Saline (G0 group) administered equivalently served as a control. **h** Tumor growth profiles (left panel) and tumor growth curves for each mouse (right panel) after treatment with the strains in (**g**). *n*=7. **i** Curves showing the survival of the mice after tumor cell inoculation. The mice were euthanized when they reached the humane endpoint. *n*=7. Data are presented as mean±SD. ^⁎^*P*<0.05, ^⁎⁎⁎^*P*<0.001, ^⁎⁎⁎⁎^*P*<0.0001, ns non-significant. EPS. Extracellular polysaccharide; CFU. Colony-forming unit; AISI. Attenuated *Salmonella* Δ*htrA*::*luxI*-VNP20009 strain; ST. SpyTag; AISI-DF. Attenuated *Salmonella* Δ*fljB*&Δ*fliC*-VNP20009; B. Blank; H. HtrA; Tnf. Tumor necrosis factor; ROS. Reactive oxygen species; NO. Nitric oxide; MYD88. Myeloid differentiation primary response 88; p-JNK. Phosphorylated c-Jun N-terminal kinase; JNK. c-Jun N-terminal kinase; p-ERK1/2. Phosphorylated extracellular signal-regulated kinase 1/2; ERK1. Extracellular signal-regulated kinase 1; p-IκB. Phosphorylated Inhibitor of κB; IκB. Inhibitor of κB; MACS. Macrophages; GFP. Green fluorescent protein; SC. SpyCatcherΔ.

We next engineered the AISI-pLuxI-HtrA-ST strain (abbreviated as AISI-H-ST), in which QS triggers HtrA protein expression (Fig. 4**a**). The AISI-ST strain transfected with an empty plasmid, denoted as AISI-B-ST, served as the blank control. Western blotting analysis confirmed HtrA expression in the AISI-H-ST strain (Fig. 4**b**). The HtrA protein indirectly increases bacterial EPS levels via the HtrA-Lon-RcsA axis [32]. Accordingly, EPS levels were significantly higher for the AISI-H-ST strain than for the AISI-B-ST strain (Fig. 4**c**). This increase in EPS levels increased both the adhesion and the thickness of the EPS layer of the AISI-H-ST strain (Fig. 4**d, e**). These findings confirm that QS-mediated HtrA re-expression increases EPS levels in the AISI-ST strain without affecting bacterial growth (Additional file 1**:** Fig. S6d). EPS can directly activate MACS through the TLR4-nuclear factor κB(NF-κB) signaling pathway [39]. Compared with the AISI-B-ST strain, the AISI-H-ST strain, which produced more EPS, more effectively induced the polarization of M0-type MACS to the antitumor M1 type (Fig. 4**f**). The EPS content of the AISI-ST strain and its ability to induce M0-type MACS to polarize to the M1 type are similar to those of the classic anticancer bacterium *Salmonella typhimurium* VNP20009 (Fig. 5**a, b**). No significant differences were observed in the expression of representative flagella-associated genes between the AISI-B-ST strain and the AISI-H-ST strain (Fig. 5**c**), suggesting that the AISI-H-ST strain significantly enhances the antitumor activity of MACS, potentially through the EPS-mediated classical TLR4 signaling pathway instead of the flagella-mediated TLR5 pathway [39] (Fig. 5**d**). Inhibition of TLR4 signal transduction significantly weakened the ability of the AISI-H-ST strain to polarize MACS to the M1 phenotype (Fig. 5**d, e**). The indirect coculture of MACS activated by the AISI-H-ST strain with tumor cells significantly inhibited tumor cell growth (Fig. 5**f**). We further tested the AISI-H-ST/SC-RGD×4 strain (AISI-H-ST strain conjugated with SC-RGD×4) in a B16-F10 murine melanoma model, alongside saline, AISI-B-ST/SC-RGD, and AISI-H-ST/SC-RGE as controls (Fig. 5**g**). Both the AISI-H-ST/SC-RGE and the AISI-H-ST/SC-RGD×4 strains produced notable antitumor effects, in contrast to the inactive AISI-B-ST/SC-RGD×4 strain (Fig. 5**h**), underscoring that QS-based HtrA re-expression increases the anticancer potential of the strains. The anticancer efficacy of the AISI-H-ST/SC-RGD×4 strain was particularly pronounced because of its faster intratumoral enrichment (Fig. 5**h**, Fig. 2). Compared with saline treatment, treatment with the AISI-H-ST/SC-RGD×4 strain also significantly prolonged the survival of tumor-bearing mice (Fig. 5**i**), confirming that the specific restoration of HtrA expression by the AISI-H-ST/SC-RGD×4 strain via QS enhances the anticancer efficacy of the engineered strain.

### Tumor-specific HtrA and PD1nb expression induced by the AISI-ST/SC-RGD×4 strain achieves potent antitumor effects

**Fig. 6:**
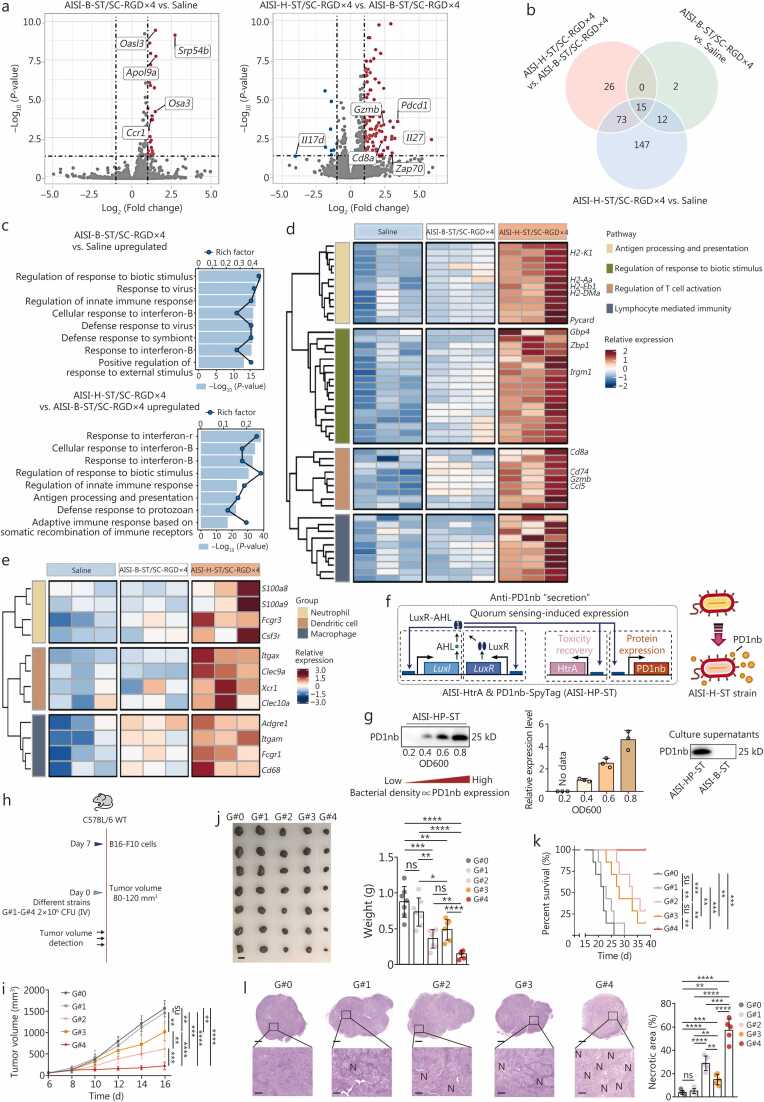
**The engineered AISI-HP-ST/SC-RGD×4 strain, which expresses HtrA and secretes PD1nb, simultaneously achieves potent antitumor efficacy**. **a** Volcano plot illustrating changes in gene expression in B16-F10 tumor tissues after treatment with saline, the AISI-B-ST/SC-RGD×4 strain, or the AISI-H-ST/SC-RGD×4 strain. **b** Venn diagram showing the overlap of upregulated genes among the different groups. **c** Bar plot showing the results of the Gene Ontology (GO) enrichment analysis of the upregulated genes among the different groups. **d** The heatmap illustrating the expression of significantly upregulated representative genes in immune-related pathways in the different groups. **e** Expression of some marker genes for macrophages, neutrophils, and dendritic cells in the different groups. **f** Circuit schematic of the engineered AISI-HP-ST strain (AISI-HtrA&PD1nb-ST) based on AISI-ST chassis bacteria. HtrA expression and PD1nb secretion are regulated by the *LuxI* promoter. **g** Immunoblot analysis of the relationship between the bacterial density and PD1nb expression *in vitro*. The immunoblot on the bar graph shows the content of secreted PD1nb in the supernatant of the strain at OD600=0.8. **h** Schematic of the different strains used for antitumor therapy, including AISI-B-ST/SC-RGD×4 (G#1 group), AISI-HtrA-ST/SC-RGD×4 (G#2 group), AISI-PD1nb-ST/SC-RGD×4 (G#3 group), and AISI-HtrA&PD1nb-ST/SC-RGD×4 (G#4 group). Saline (G#0 group) was used as the blank control. **i** Tumor growth profiles after the different treatments in (**h**). *n*=7. **j** Tumors were photographed (left panel) and weighed (right panel) on day 16 after the administration of the treatments to the groups in (i). Scale bar=10 mm. **k** Survival curves for the mice in (**h**). The mice were euthanized when they reached the humane endpoint. **l** The tumor necrosis area as a percentage of the total tumor area in the different groups was measured via H&E staining on day 3. Tumors of similar size were selected to avoid interference caused by differences in vascularity and hypoxic areas due to the tumor volume. Five visual fields were randomly selected, and the necrotic area (labeled N) in each visual field was quantified. Scale bar=10 μm for full tumor sections and 200 μm for enlarged tumor sections. The data are presented as the mean±SD. ^⁎^*P*<0.05, ^⁎⁎^*P*<0.01, ^⁎⁎⁎^*P*<0.001, ^⁎⁎⁎⁎^*P*<0.0001, ns non-significant. AISI. Attenuated *Salmonella* Δ*htrA*::*luxI*-VNP20009 strains; ST. SpyTag; SC. SpyCatcher; RGD. Arginine-glycine-aspartic acid; HtrA. High-temperature requirement A; PD1. Programmed cell death protein 1; B. Blank; H. HtrA; HP. HtrA and PD1nb; AHL. N-acyl homoserine lactone; WT. Wild type.

Following treatment with the AISI-H-ST/SC-RGD×4 strain, notable changes were observed in the tumor microenvironment (TME). Flow cytometry analyses revealed a marked increase in the number of necrotic cells within the tumors of the group treated with the AISI-H-ST/SC-RGD×4 strain compared with those of the saline- or AISI-B-ST/SC-RGD×4**-**treated groups **(**Additional file 1**:** Fig. S7a). Additionally, this group exhibited a significant increase in the number of infiltrating intratumoral immune cells by day 3 after treatment (Additional file 1**:** Fig. S7b). Consequently, tumor tissues on the day 3 were selected for total RNA sequencing. The comparative analysis revealed that the expression of most genes was upregulated in the AISI-B-ST/SC-RGD×4 group compared with that in the saline group and was similarly increased in the AISI-H-ST/SC-RGD×4 group (Fig. 6**a, b**). The Gene Ontology (GO) analysis revealed that compared with the AISI-B-ST/SC-RGD×4 strain, the AISI-H-ST/SC-RGD×4 strain more strongly upregulated immune response-related biological processes (Fig. 6**c, d**). Furthermore, the expression of anticancer genes in key intratumoral immune cells, including MACS, neutrophils, and dendritic cells (DCs), was substantially higher in the AISI-H-ST/SC-RGD×4 group than in both the saline and AISI-B-ST/SC-RGD×4 groups (Fig. 6**e**). These findings underscore potent intratumoral immune activation by the AISI-H-ST/SC-RGD×4 strain, which correlates with its enhanced antitumor efficacy and is attributed to tumor-specific HtrA expression.

The MetaCore gene network analysis revealed that the AISI-H-ST/SC-RGD×4 strain induced stronger antigen presentation and T cell activation, suggesting a potential synergistic effect with immune checkpoint blockade therapy (Additional file 1**:** Fig. S7c). Consequently, we developed the AISI-HtrA&PD1nb-ST/SC-RGD×4 strain (hereafter referred to as AISI-HP-ST/SC-RGD×4), which expresses the HtrA protein and secretes the anti-PD1nb at high bacterial densities (Fig. 6**f**). Western blotting analysis confirmed an increase in PD1nb expression levels proportional to the strain density, followed by the pelB signaling peptide-mediated secretion into the culture medium via the Sec-dependent secretion pathway (Fig. 6**g**). The expression of PD1nb did not affect the simultaneous expression of HtrA at high density in the strain (Additional file 1**:** Fig. S8a). This secreted PD1nb blocks the PD1-PDL1 pathway, reversing immunosuppression and suppressing tumor growth [40]. In B16-F10 tumor-bearing mice, compared with the single-expressing or nonexpressing controls, the AISI-HP-ST/SC-RGD×4 strain induced superior tumor suppression. Tumor growth monitoring revealed significantly reduced tumor volumes and weights in the AISI-HP-ST/SC-RGD×4 group, with a mean tumor weight of 0.146 g, which is significantly lower than those in the other groups and equivalent to 16.7% of that in the saline group (Fig. 6**h-j;** Additional file 1**:** Fig. S8b). Moreover, while fewer than 50% of the mice in the AISI-H-ST/SC-RGD×4 and AISI-P-ST/SC-RGD×4 (AISI-PD1nb-ST/SC-RGD×4) groups survived beyond 35 d, 100% of those in the AISI-HP-ST/SC-RGD×4 group survived (Fig. 6**k**). Notably, the tumor necrotic area exceeded 60% within 3 d after treatment with the AISI-HP-ST/SC-RGD×4 strain, which was substantially greater than that of any other treatment (Fig. 6**l**). Collectively, these results highlight the robust anticancer efficacy of the AISI-HP-ST/SC-RGD×4 strain through the concurrent expression of HtrA and PD1nb.

### The AISI-HP-ST/SC-RGD×4 strain promotes antitumor immunity through the synergistic effects of HtrA and PD1nb

**Fig. 7:**
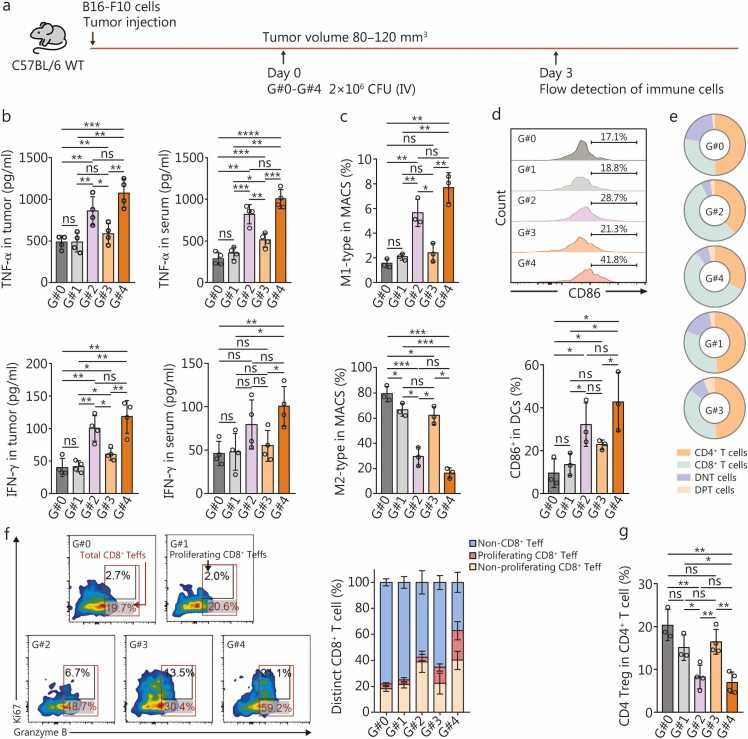
**The engineered AISI-HP-ST/SC-RGD×4 strain effectively activates antitumor immunity**. **a** Schematic of the different strains used for the analysis of the potential antitumor mechanism, including AISI-B-ST/SC-RGD×4 (G#1 group), AISI-H-ST/SC-RGD×4 (G#2 group), AISI-P-ST/SC-RGD×4 (G#3 group), and AISI-HP-ST/SC-RGD×4 (G#4 group). Saline (G#0 group) was used as the blank control. Cytokines and immune cells were detected on Day 3 after the different treatments in (**b-g**). **b** The concentrations of the antitumor cytokines TNF-α (left top panel) and IFN-γ (left bottom panel) in tumors as well as antitumor cytokines TNF-α (right top panel) and IFN-γ (right bottom panel) in serum were determined. *n*=4. **c** Percentages of M1-type antitumor tumor infiltrating macrophages (top panel) and M2-type protumor infiltrating macrophages (bottom panel) in tumors. **d** A representative flow cytometry plot (top panel) and a bar chart (bottom panel) show the change in the percentage of mature dendritic cells (DCs) (CD11c^+^MHCII^+^ CD86^+^) in tumors. **e** Proportions of different T lymphocytes, including CD4^+^ T cells, CD8^+^ T cells, CD4^+^CD8^+^ T cells (double-positive cells, DPT cells), and CD4^-^CD8^-^ T cells (double-negative cells, DNT cells), among the total tumor-infiltrating lymphocytes in tumors. **f** Percentages of non-CD8^+^ Teffs (CD8^+^GzmB^-^), proliferating CD8^+^ Teffs (CD8^+^GzmB^+^Ki67^+^), and non-proliferating CD8^+^ Teffs (CD8^+^GzmB^+^Ki67^-^) among total CD8^+^ T cells in tumors. **g** Percentage of protumor tumor-infiltrating CD4^+^ Tregs (CD4^+^Foxp3^+^) in tumors. The data are reported as the mean±SD. ^⁎^*P*<0.05, ^⁎⁎^*P*<0.01, ^⁎⁎⁎^*P*<0.001, ^⁎⁎⁎⁎^*P*<0.0001, ns non-significant. AISI. Attenuated *Salmonella* Δ*htrA*::*luxI*-VNP20009 strains; ST. SpyTag; SC. SpyCatcherΔ; RGD. Arginine-glycine-aspartic acid; HtrA. High-temperature requirement A; PD1. Programmed cell death protein 1; B. Blank; H. HtrA; HP. HtrA and PD1nb; WT. Wild type; CFU. Colony-forming unit; IV. Intravenous; TNF-α. Tumor necrosis factor-α; IFN-γ. Interferon-γ; MACS. Macrophages; Teffs. Effector T cells; Tregs. Regulatory T cells; CD4^+^ T cells. Cluster of differentiation 4-positive T lymphocyte; CD8^+^ T cells. Cluster of differentiation 8-positive T lymphocyte; DNT. Double-negative T cells; DPT. Double-positive T cells.

We conducted a detailed analysis of the TME in B16-F10 tumor-bearing mice at 72 h after administration to explore the antitumor mechanisms mediated by the engineered strain AISI-HP-ST/SC-RGD×4 (Fig. 7**a**). Cytokine profiling via ELISA revealed that, compared with the control strain AISI-B-ST/SC-RGD×4, the AISI-H-ST/SC-RGD×4 strain significantly increased the secretion of critical antitumor cytokines, including TNF-αand IFN-γ, within both the tumor tissue and systemic circulation (Fig. 7**b**). Although the level of cytokines in the AISI-HP-ST/SC-RGD×4 group tended to increase, the difference did not reach statistical significance compared with that in the AISI-H-ST/SC-RGD×4 group (Fig. 7**b**). The flow cytometry analysis of tumor-associated macrophages (TAMs) revealed a marked shift in polarization dynamics. Both the AISI-H-ST/SC-RGD×4 and the AISI-HP-ST/SC-RGD×4 strains significantly increased the proportion of antitumor M1-type TAMs while concurrently reducing the proportion of protumor M2-type TAMs (Fig. 7**c**; Additional file 1**:** Fig. S9). Quantitatively, the AISI-HP-ST/SC-RGD×4 group presented a 3.70-fold increase in the number of M1-type TAMs and a 24.8% reduction in M2-type TAMs to 24.8% of the level observed in the AISI-B-ST/SC-RGD×4 group (Fig. 7**c**). Furthermore, the expression of HtrA and PD1nb by these strains was associated with enhanced DC maturation, as evidenced by the upregulation of the surface markers CD86 (Fig. 7**d**). Given the well-established role of mature DCs in priming CD8^+^ T cell responses [41], we observed a substantial increase in the proportion of effector CD8^+^ T cells [effector T cells (Teffs)] in the AISI-HP-ST/SC-RGD×4 group. Specifically, the Teff population in the AISI-HP-ST/SC-RGD×4 group was 2.53-fold higher than that in the AISI-B-ST/SC-RGD×4 group, whereas the Teff populations in the AISI-H-ST/SC-RGD×4 and AISI-P-ST/SC-RGD×4 groups were 1.70-fold and 1.39-fold higher, respectively (Fig. 7**e, f**). These findings underscore the multifaceted antitumor activity of the AISI-HP-ST/SC-RGD×4 strain, which orchestrates a favorable immune milieu by promoting M1-type TAM polarization, enhancing DC maturation, and amplifying CD8^+^ T cell effector functions. Although the proportion of CD8 Teffs among the total CD8^+^ T cells was similar in the AISI-H-ST/SC-RGD×4 and AISI-P-ST/SC-RGD×4 groups, the latter presented a higher proportion of proliferating CD8^+^ Teffs (approximately 12.5%), whereas the proportion of the former group (approximately 3.3%) was similar to that of the AISI-B-ST/SC-RGD×4 group (approximately 3.7%). These findings suggested that HtrA-expressing bacteria strongly activated CD8^+^ T cells but failed to trigger their proliferation. In contrast, PD1nb-secreting bacteria, which exhibit relatively weak CD8^+^ T cell activation, effectively induced cell proliferation (Fig. 7**f**). A similar phenomenon has been reported previously [40], [42]. In the AISI-HP-ST/SC-RGD×4 group, in which HtrA and PD1nb were simultaneously produced, the proportion of CD8^+^ Teffs was significantly increased. More importantly, about 36.5% of these cells were proliferative, a value that was 4.73 times higher than that of the AISI-H-ST/SC-RGD×4 group (Fig. 7**f**). The simultaneous expression of PD1nb and HtrA by the AISI-ST/SC-RGD×4 strain indeed led to synergistic antitumor immune activation. In addition, the HtrA-expressing strain reduced the proportion of Tregs among CD4^+^ T cells, whereas the PD1nb-expressing strain did not (Fig. 7**g**). Overall, the octopus-inspired novel engineered bacterium AISI-HP-ST/SC-RGD×4 (named OITE-bacteria) robustly activated antitumor immunity, which may have contributed to its potent anticancer function.

## Discussion

**Fig. 8:**
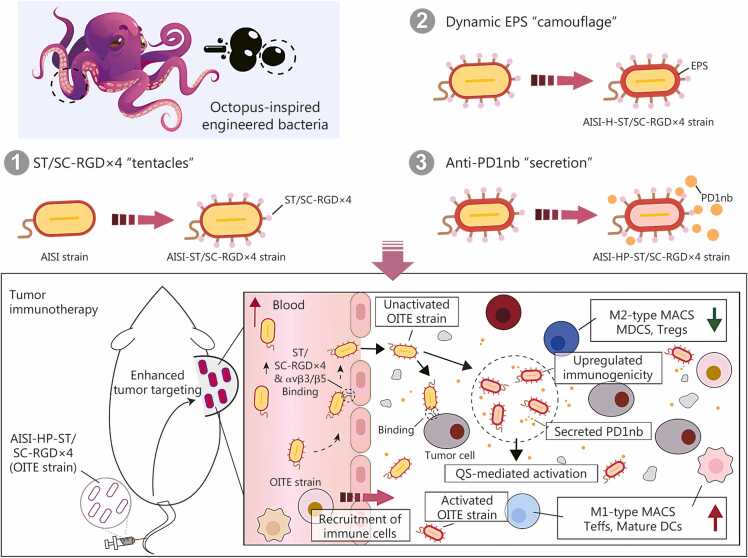
**Schematic of the construction and therapeutic mechanism of the octopus-inspired triple-engineered bacteria (OITE strain)**. The antitumor attenuated *Salmonella typhimurium* AISI strain was triple-engineered. (1) ST/SC-RGD×4 “tentacles”-Surface modification: the ST sequence was expressed within the external third loop of OmpA in the AISI strain, resulting in the AISI-ST strain. The ST protein was coexpressed with OmpA (OmpA-ST fusion protein) localized to the bacterial outer membrane. An incubation with SC-RGD×4 protein (SC-RGD×4) led to the formation of AISI-ST/SC-RGD×4 strain (AISI-ST/SC-RGD×4) through ST-SC-mediated covalent conjugation. (2) Dynamic EPS “camouflage”-Immunoactivation engineering: the AISI-H-ST strain was created by introducing the quorum-sensing (QS) promoter pLuxI to control HtrA expression into AISI-ST strain, which specifically increased HtrA-mediated extracellular polysaccharide (EPS) production to amplify bacteria-mediated immune activation. (3) Anti-PD1nanobody (PD1nb) “secretion”-Checkpoint blockade engineering: further programming of AISI-H-ST strain enabled QS-triggered anti-PD1nb secretion, generating the AISI-HP-ST strain. The incubation of AISI-HP-ST strain with SC-RGD×4 protein finally produced the OITE strain (AISI-HP-ST/SC-RGD×4). The intravenously administered OITE strain highly selectively accumulates in tumors through RGD-αvβ3 integrin interactions, with subsequent bacterial proliferation initiating two therapeutic actions: HtrA-mediated immune activation via increasing immune cells infiltration and activation, and secreted PD1nb-based blockade of PD-1/PD-L1 immunosuppressive signaling. OITE. Octopus-inspired, triple-engineered bacterium; AISI. Attenuated *Salmonella* Δ*htrA*::*luxI*-VNP20009 strains; ST. SpyTag; SC. SpyCatcherΔ; RGD. Arginine-glycine-aspartic acid; HtrA. High-temperature requirement A; PD1. Programmed cell death protein 1; H. HtrA; HP. HtrA and PD1nb; AHL. N-acyl homoserine lactone; MACS. Macrophages; Teffs. Effector T cells; Tregs. Regulatory T cells.

The effective colonization of bacteria in various tumor types is well established. Once bacteria reach tumors, they can proliferate because of the tumor-specific microenvironment, which is characterized by hypoxia and immunosuppression [2], [3]. Thus, the rapid “arrival, recognition, and adhesion” of bacteria to tumors following administration is crucial for their timely and efficient colonization. Several strategies have been employed to increase bacterial targeting to tumors, including the genetic modification of strains [43] and the use of immune cells as delivery vehicles [6], [9], [44]. Clearly, the successful clinical translation of bacterial cancer therapies requires the development of more precise and potent treatments. The addition of tumor-targeting RGD peptides to the bacterial surface has been shown to enhance tumor enrichment [20]. Compared with monomeric RGD peptides, multivalent RGD peptides, such as triple and quadruple variants, demonstrate significantly greater tumor-targeting and enrichment capabilities. However, these multimers are limited to chemical synthesis and cannot be naturally produced by bacteria. Furthermore, directing complex proteins to specific cellular locations, such as the bacterial outer membrane, remains technically challenging and often leads to protein misfolding. In this study, we employed a SC-ST-based covalent conjugation strategy to attach multivalent RGD peptides to the bacterial surface, thereby enhancing tumor targeting (Fig. 8). This clicking approach enables bacteria to carry substantially higher densities of RGD peptides, resulting in more than a 3-fold improvement in tumor targeting compared with bacteria modified with a single RGD peptide. Importantly, this conjugation strategy expands the scope for more diverse biological and chemical modifications of bacterial strains. We validated the stable covalent interaction between the surface-displayed ST on the engineered strain AISI-ST and SC using two complementary approaches, fluorescence imaging following co-incubation of AISI-ST with SC-eGFP, and Western blotting detecting the characteristic mobility shift of the ST-containing band. Together, these results confirm the successful display of SC on the bacterial surface. Future studies could further leverage mass spectrometry-based approaches to achieve more precise qualitative and quantitative characterization of SC fusion protein presentation in this modular modification strategy for personalized therapeutic applications, thereby extending both its depth and breadth of applicability.

Unlike previous studies in which engineered bacteria simply expressed secreted anticancer molecules for tumor therapy, our approach involves three remodeling steps (Fig. 8). These modifications not only increase the intratumoral enrichment of the strain but also restore intratumoral-specific immunogenicity. When engineered bacteria reach high densities within tumors or when local colonies are spatially close enough, the self-induced signaling molecules they accumulate can reach threshold levels, thereby activating the QS system and initiating specific response characteristics [11], [37]. By controlling protein expression with a QS system, we achieved more precise and durable expression and release of therapeutic proteins within the tumor. We confirmed that robustly activated bioluminescent signals driven by the QS system were detectable within tumors. These signals emerged as early as 18 h post-administration and increased progressively over time (Additional file 1**:** Fig. S6), consistent with previous reports [32]. We also showed that bacteria-mediated restoration of intratumoral immunogenicity via EPSs is crucial for the efficacy of anti-PD1nb-mediated immune blockade therapy, particularly in activating CD8^+^ T cells. More importantly, the controlled elevation of bacterial EPSs within tumors preserves the excellent biosafety profile of the chassis strain [32]. These findings highlight the synergistic anticancer efficacy of combining bacterial therapy with immune blockade therapy. Further investigation into this synergistic mode of action could guide more refined bacterial modifications and combination strategies.

Given the differential sensitivity of distinct tumor types to various immune checkpoint inhibitors [45], the modularity of this engineered strain allows it to be readily adapted for the expression of other immune checkpoint-blocking nanobodies, such as anti-PD-L1 and anti-CTLA-4, in addition to anti-PD-1 [46], thereby enabling broader and more flexible clinical applications. Previous study has shown that bacterial infections can induce granuloma-like structures whose spatial organization may shield the infiltration patterns and distribution of immune cells in tissues [47]. Therefore, further investigations of whether engineered strains may similarly shield the infiltration of immune cells in the TME to some extent are worthwhile and could aid in the targeted development of engineered strains with enhanced anticancer efficacy. In general, bacteria residing within tumors can persist for several weeks, whereas bacteria localized to healthy organs are rapidly cleared [6], [32]. In clinical trials of bacteria-based anticancer therapies, appropriate dosing did not lead to significant abnormalities in serum inflammatory markers, with only mild adverse reactions observed, and no substantial systemic toxicity or risk of cytokine storm reported [2], [13]. Together, these findings further support the favorable biosafety profile and clinical translation potential of bacterial therapies.

In the future, development of this modification strategy should focus on improving scalability and validating its efficacy in complex biological systems. Here, a dynamic population-sensing approach was implemented to restore bacterial immunogenicity and, in parallel, to trigger anti-PD1 nanobody (anti-PD1nb) secretion for tumor cotreatment. As reported previously, this prodrug delivery-like initiation strategy is well recognized for its specificity [7]. Based on this established specificity, a tumor-specific increase in bacterial EPS content is expected to further enhance immunogenicity by promoting TLR-dependent immune cell activation and improving bacterial stress resistance, thereby facilitating a more robust conversion of “cold tumors” into “hot tumors” [32], [39]. Nonetheless, consistent with other regulated expression systems, activation of engineered strains can still permit leakage from the intratumoral microenvironment into the bloodstream, potentially leading to adverse effects [48]. One promising strategy to mitigate this risk is the development of safer chassis strains. Benefiting from the favorable biocompatibility of the AISI strain, no significant adverse effects were observed for the triple-modified engineered strain in the *in vivo* experiments. Implementing genetic edits to enable the spontaneous self-inactivation of leaked strains in response to healthy environmental cues represents another reference-worthy and highly attractive strategy. Although the bacterial origin of the SpyCatcher protein has raised concerns regarding potential immunogenicity, previous studies have reported only limited immune responses in therapeutic contexts [29], [49]. Nonetheless, continued evaluation of immunogenicity will remain essential in future translational studies. Moreover, although the triple modification strategy we describe has been effectively verified in mouse models, the ability of this initiation system to maintain high tumor specificity and responsiveness in the complex clinical milieu remains to be validated. Alternatives, other induced expression systems, such as the basis of artificially added inducers [e.g., isopropyl β-D-1-thiogalactopyranoside (IPTG)], exogenous stimuli (e.g., heat), or clinically validated tumor-specific inducers, could be considered [48]. The administration methods, dosages, and applicable cancer types for these strains, which are suitable for clinical use in patients, also need to be further clarified to more effectively achieve their translation from mice to humans. This issue is particularly important given that integrin αvβ3 is also highly expressed at wound-healing sites and during neovascularization [50]. The presence of αvβ3 in these non-tumoral tissues introduces a potential risk of off-target binding by engineered bacteria. Therefore, application of this strain in postoperative cancer patients, individuals with traumatic injuries, or patients exhibiting pathological angiogenesis, such as those with diabetes, warrants careful consideration.

The “suckers” described here represent a modular strategy that offers significantly greater flexibility and versatility than traditional single-use biological display approaches. We selected the tumor-targeting peptide RGD due to its established applications in clinical tumor tracer radiolabeling and drug conjugation. Beyond RGD, the expression of fusion proteins, such as integrins or antibody fragments targeting tumor antigens including CD20 [17] and carcinoembryonic antigen [51] that are covalently spliced to ST-labeled bacteria, either individually or in combination, is also feasible for bacterial targeting. Moreover, based on the premise that the patient’s tumor lesion pathology or sequencing results are clear, rapid preparation of personalized recombinant strains that are more suitable for clinical patients is possible. For example, targeted therapy for solid tumors such as breast cancer (with high HER2 expression) [52] is used in clinical practice. This personalized clicking strategy could help this bacterial therapy navigate more complex clinical settings and expedite the clinical translation of bacterial therapies. We confirmed that bacteria were able to persistently colonize tumors for more than 10 d. Although prolonged bacterial presence may induce antibody production, few studies have reported that such antibody responses substantially compromise the antitumor efficacy of bacterial therapies in preclinical models [3], [37]. Nevertheless, further evaluation in future clinical studies remains necessary. Engineered bacteria often increase the sensitivity of tumor cells to chemotherapeutic agents and protein drugs and remodel the tumor tissue structure, effectively inducing the infiltration of immune cells [2], [18]. Thus, such strains also hold promise for combination use with other traditional (e.g., radiotherapy and chemotherapy [53]) or emerging (e.g., CAR-T cell therapy [54]) tumor treatment modalities to synergistically achieve more potent antitumor efficacy, which warrants further investigation. This study evaluated the targeting performance and therapeutic efficacy of the engineered bacteria in female tumor-bearing mice. However, potential sex-dependent differences in efficacy or toxicity were not examined. Future studies incorporating male animals will be important to clarify the influence of sex on treatment outcomes and to inform the optimization of dosing strategies in clinical settings based on sex-specific patient characteristics.

## Conclusions

In this study, we developed an octopus-inspired triple-engineered bacterium, OITE-bacteria. This construct incorporates three key modifications: 1) the AISI-ST strain expresses ST on its outer membrane, enabling rapid decoration with RGD peptides by simple 1 h incubation with SC-RGD×4 (first remodeling); 2) the AISI-H-ST strain, derived from AISI-ST, uses QS to drive HtrA expression (second remodeling); and 3) the AISI-HP-ST strain, based on AISI-H-ST, employs QS to induce anti-PD1nb expression (third remodeling). The RGD×4 peptide promotes rapid and efficient intratumoral enrichment, while tumor-specific HtrA expression reduces off-target toxicity and enhances immune reactivation within tumors. In addition, anti-PD1nb expression and release further strengthen bacterium-mediated antitumor immunity. By integrating bacterial surface display for tumor anchoring with QS-controlled therapeutic protein expression, this platform enables intelligent, sequential activation from targeting to treatment. Its standardized modular design also allows rapid replacement of targeting motifs or therapeutic payloads through simple cloning, improving development efficiency over conventional approaches. This work provides a rationally designed prototype platform with improved targeting precision, safety, and programmability for next-generation low-toxicity intelligent live bacterial therapies.

## Abbreviations

AISI-ST: Attenuated *Salmonella* Δ*htrA*::*luxI*-VNP20009 strain expressing OmpA-SpyTag

Asp: Aspartic acid

BSA: Bovine serum albumin

CD: Cluster of differentiation

CFU: Colony-forming unit

DAPI: 4’,6-diamidino-2-phenylindole

DC: Dendritic cell

eGFP: Enhanced green fluorescent protein

ELISA: Enzyme-linked immunosorbent assay

EPS: Extracellular polysaccharide

ERK1: Extracellular signal-regulated kinase 1

FimH: Type 1 fimbriae D-mannose-specific adhesin

HtrA: High-temperature requirement A

IFN-γ: Interferon-γ

IκB: Inhibitor of κB

IV: Intravenous

JNK: c-Jun N-terminal kinase

LB: Luria-Bertani

Lys: Lysine

MACS: Macrophages

MHC: Major histocompatibility complex

MYD88: Myeloid differentiation primary response 88

NO: Nitric oxide

OMPs: Outer membrane proteins

p-ERK1/2: Phosphorylated extracellular signal-regulated kinase 1/2

p-IκB: Phosphorylated Inhibitor of κB

p-JNK: Phosphorylated c-Jun N-terminal kinase

PBS: Phosphate-buffered saline

PCR: Polymerase chain reaction

PD1: Programmed cell death protein 1

PD1nb: PD1 nanobody

QS: Quorum-sensing

RFP: Red fluorescent protein

RGD: Arginine-glycine-aspartic acid

RGE: Arginine-glycine-glutamic acid

ROS: Reactive oxygen species

SDs: Standard deviations

ST/SC: SpyTag-SpyCatcherΔ

TAMs: Tumor-associated macrophages

Teffs: Effector T cells

TLR4: Toll-like receptor 4

TME: The tumor microenvironment

TNF-α: Tumor necrosis factor-α

Tregs: Regulatory T cells

## Ethics approval and consent to participate

All procedures were conducted following ethical guidelines and were approved by the Institutional Animal Care and Use Committee (IACUC) of Nanjing University (IACUC-2003167).

## Funding

This work was supported in part by the National Natural Science Foundation of China (82303774, 82130106), the Natural Science Foundation of Jiangsu Province (BK20243001, BG2024026, BK20230165, BE2023695, BY20241051), and the Changzhou Municipal Department of Science and Technology (CE20246001, CJ20250023), China.

## Data Availability

Any data associated with this study are available from the corresponding author upon reasonable request.
