# Supplementary material for: Octopus-inspired engineered bacteria with a plug-and-play surface display system achieves enhanced tumor-specific colonization and antitumor immunity
Source: Mil Med Res. 2026 Apr 27;13(1):100030. doi: 10.1016/j.mmr.2026.100030 (PMC13138155; doi:10.1016/j.mmr.2026.100030)
Supplement: Supplementary file 1 — Supplementary material [file mmc1.pdf]

Table S1 The sequences of the primers used for RT-qPCR

Gene	Primer sequence	Primer length (bp)
β-actin	Forward: 5′-GCACCACACCTTCTACAATGAG-3′	22
	Reverse: 5′-TTGGCATAGAGGTCTTTACGGA-3′	22
Ccl5	Forward: 5′-GCTGCTTTGCCTACCTCTCC-3′	20
	Forward: 5′-TCGAGTGACAAACACGACTGC-3′	21
Ccl2	Forward: 5′-TTAAAAACCTGGATCGGAACCAA-3′	23
	Forward: 5′-GCATTAGCTTCAGATTTACGGGT-3′	23
Tnf	Forward: 5′-GACGTGGAACTGGCAGAAGAG-3′	21
	Forward: 5′-TTGGTGGTTTGTGAGTGTGAG-3′	21
Cd68	Forward: 5′-TGTCTGATCTTGCTAGGACCG-3′	21
	Reverse: 5′-GAGAGTAACGGCCTTTTTGTGA-3′	22
Cd80	Forward: 5′-TGCTGCTGATTCGTCTTTCAC-3′	21
	Reverse: 5′-GAGGAGAGTTGTAACGGCAAG-3′	21
Cd86	Forward: 5′-GGTGGCCTTTTTGACACTCTC-3′	21
	Reverse: 5′-TGAGGTAGAGGTAGGAGGATCTT-3′	23
Il1b	Forward: 5′-GCAACTGTTCCTGAACTCAACT-3′	22
	Forward: 5′-ATCTTTTGGGGTCCGTCAACT-3′	21
Il6	Forward: 5′-TAGTCCTTCCTACCCCAATTTCC-3′	23
	Forward: 5′-TTGGTCCTTAGCCACTCCTTC-3′	21
Il8	Forward: 5′-CAAGGCTGGTCCATGCTCC-3′	19
	Reverse: 5′-TGCTATCACTTCCTTTCTGTTGC-3′	23
Il12	Forward: 5′-TGGTTTGCCATCGTTTTGCTG-3′	21
	Reverse: 5′-ACAGGTGAGGTTCACTGTTTCT-3′	22
Il18	Forward: 5′-GACTCTTGCGTCAACTTCAAGG-3′	22
	Reverse: 5′-CAGGCTGTCTTTTGTCAACGA-3′	21
Il23a	Forward: 5′-AATAATGTGCCCCGTATCCAGT-3′	22
	Reverse: 5′-GCTCCCCTTTGAAGATGTCAG-3′	21
Ifnr	Forward: 5′-TGCCCAGCAGATCAAGAAGG-3′	20
	Reverse: 5′-TCAGGGGAAATTCCTGCACC-3′	20
FljB	Forward: 5′-GTACAGTAACCCTTGCGGCT-3′	20
	Reverse: 5′-GCTCCTGTCGCTTCATCGTA-3′	20
FliC	Forward: 5′-CGTGTCAACCTGTGCCAAAG-3′	20
	Reverse: 5′-TACGCTGCAAGTAAAGCCGA-3′	20

RANTES (Ccl5). Regulated upon activation, normal T cell expressed and secreted; MCP1 (Ccl2). Monocyte

chemoattractant protein-1; TNFα (Tnf). Tumor necrosis factor-α; CD. Cluster of differentiation; Il. Interleukin; IFNγ (Ifnr). Interferon-γ; FljB. Flagellar phase 2 gene B; FliC. Flagellar phase 2 gene C

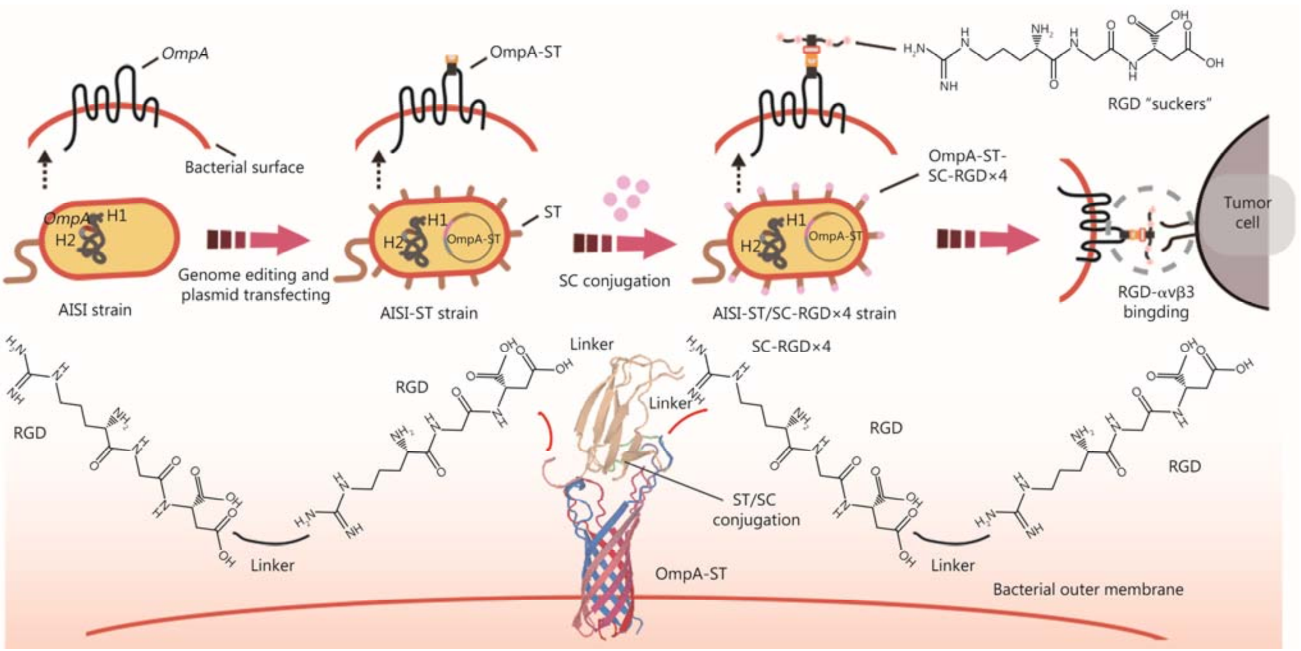

Fig. S1 Schematic illustrating the roles of the AISI-ST/SC-RGD×4 constructs. The RGD peptides on the AISI-ST/SC-RGD×4 strain specifically recognize and bind to αvβ3 integrins on tumor cells. AISI. Attenuated *Salmonella* Δ*htrA*::*luxI*-VNP20009 strain; OmpA. Outer membrane protein A; ST. SpyTag; SC. SpyCatcherΔ; RGD. Arginine-glycine-aspartic acid; ST/SC-RGD×4. SpyTag/SpyCatcherΔ-RGD×4

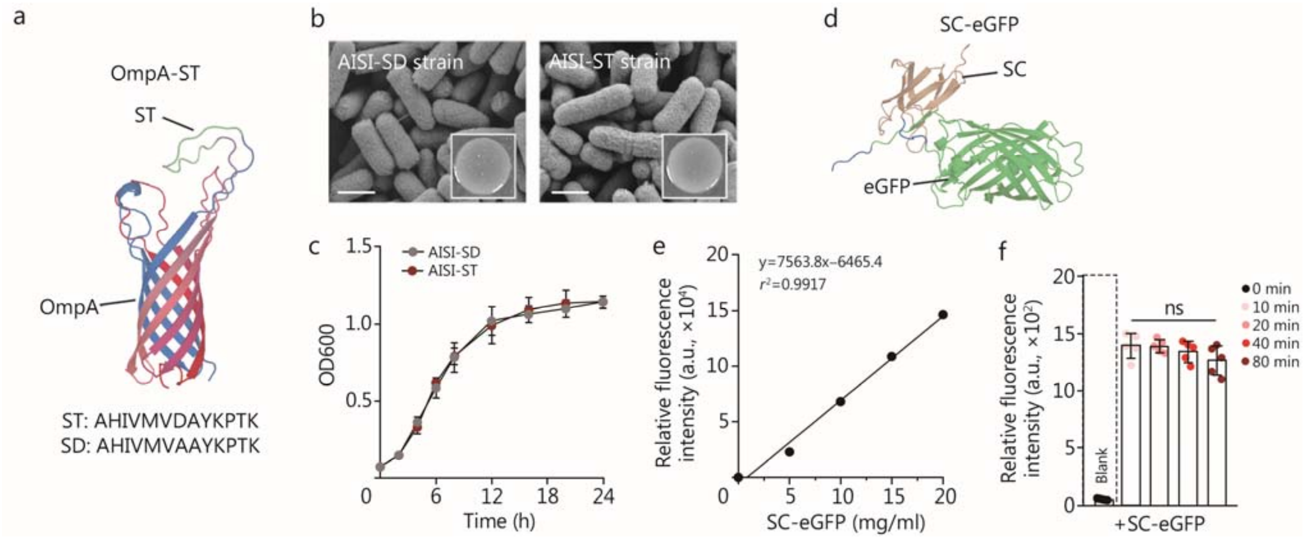

Fig. S2 Construction and evaluation of the AISI-ST strain. **a** The ST protein structure was inserted into the third transmembrane domain of OmpA, with the ST-DA (SD) protein structure serving as a blank control. **b** Representative scanning electron microscope images of the two engineered strains, AISI-SD and AISI-ST, along with magnified images of single colonies (bottom left). Scale bar=1 μm. **c** Comparison of the growth curves of AISI-SD and AISI-ST strains. *n*=3. Data are presented as means±SD. **d** Schematic representation of the molecular structure of the SC-eGFP fusion protein. **e** Relationship between SC-eGFP fusion protein concentration and fluorescence intensity. **f** Flow cytometry analysis of eGFP fluorescence intensity changes in the AISI-ST/SC-eGFP strain after continuous incubation in fetal bovine serum for different durations (0, 20, 40, 80 min). *n*=5. Data are presented as means±SD. ns. Non-significant; AISI. Attenuated *Salmonella* Δ*htrA*::*luxI*-VNP20009 strain; ST. SpyTag; SC. SpyCatcherΔ; eGFP. Enhanced green fluorescent protein; DA. Asp117. Ala mutant

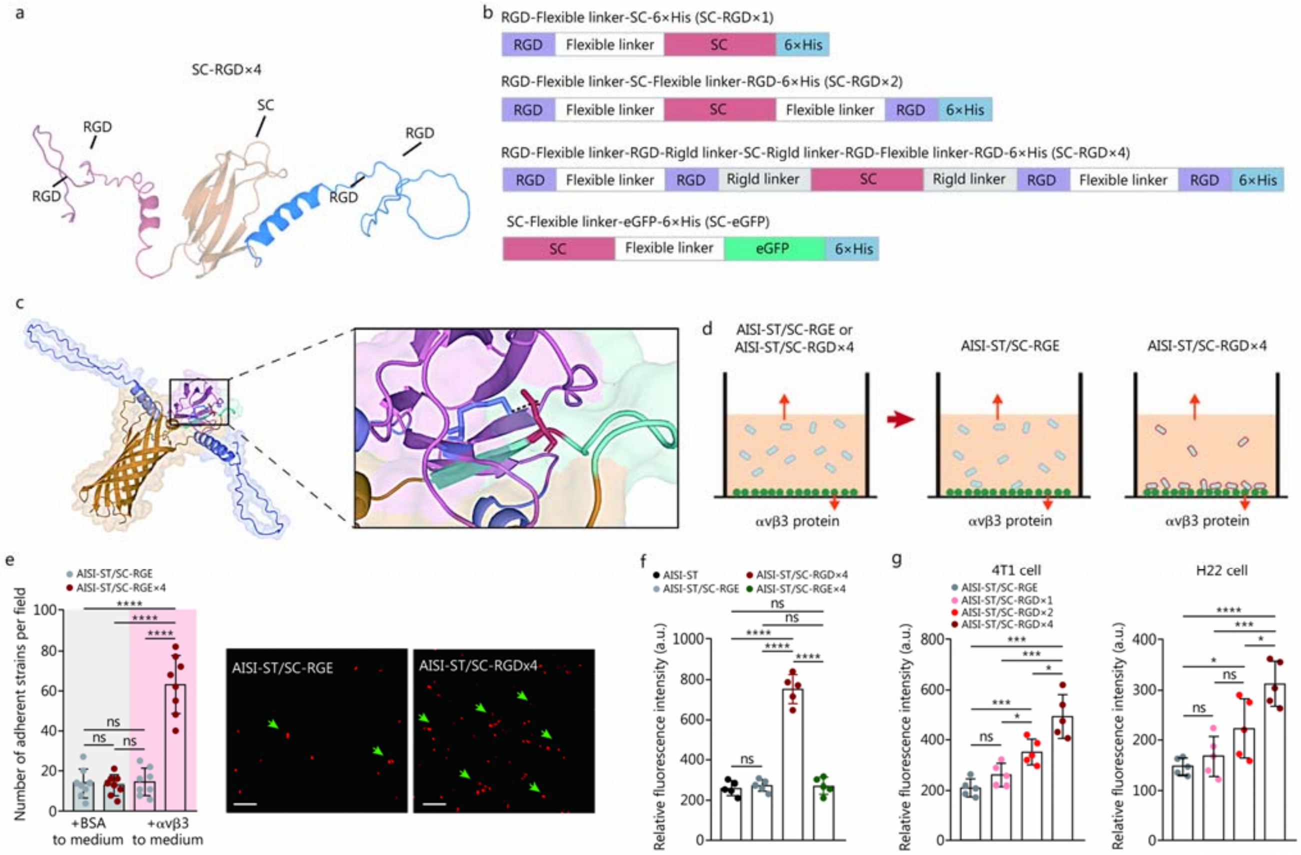

Fig. S3 Construction and evaluation of SC-RGD suckers. **a** Structural prediction of the SC-RGD×4 fusion protein. **b** Schematic representation of the engineered constructs. SC-RGD×1: consist of an RGD sequence at the N-terminus, a flexible linker (GGG GSG GGG SGG GGS), SC, and a 6×His tag. SC-RGD×2: consist of an RGD sequence at the N-terminus, an RGD sequence at the C-terminus, two flexible linkers (GGG GSG GGG SGG GGS), SC, and a 6×His tag. SC-RGD×4: consist of two RGD sequences at the N-terminus, two RGD sequences at the C-terminus, two flexible linkers (GGG GSG GGG SGG GGS), two rigid linkers (AEA AAK EAA AKE AAA KA), SC and a 6×His tag. SC-eGFP: consist of a 6×His tag, a flexible linker (GGG GS), SC, and an eGFP sequence. **c** Molecular docking simulation of the SC-RGD×4 peptide with the head domain of OmpA-ST. The purple region represents the RGD peptide, the pink region represents SC, the green region represents ST, and the yellow region represents OmpA. The enlarged partial view displays the classic interaction interface. The amino acid marked in purple is Lys (L-lysine), located on SC, and the amino acid marked in red is Asp (aspartic acid), located on ST. The two are connected through a covalent interaction. **d** Schematic of coculture of different engineered strains, including AISI-ST/SC-RGE and AISI-ST/SC-RGD×4, in dishes coated with αvβ3 protein at the bottom. A chassis-engineered strain that stably expresses red fluorescent protein (RFP) was used. The group supplemented with bovine serum albumin (BSA) at the bottom served as the control. **e** Comparison of adherent bacterial counts in random fields on the bottom of

dishes in (b). *n*=8. The AISI-ST/SC-RGD×4 strain group showed stronger bottom adhesion. Representative images of the different bacterial adhesion to αvβ3 protein were presented (right panel). Scale bars=20 μm. **f** Different engineered strains, including AISI-ST, AISI-ST/SC-RGE, AISI-ST/SC-RGD×4, and AISI-ST/SC-RGE×4, were co-incubated with B16-F10 tumor cells at an MOI of 100. Free bacteria were removed, and the total fluorescence intensity of the strains adhering to tumor cells was detected. A chassis-engineered strain that stably expresses red fluorescent protein (RFP) was used. AISI-ST/SC-RGE×4 strain did not show stronger tumor cell adhesion properties than AISI-ST/SC-RGE strain. **g** Different engineered strains, including AISI-ST/SC-RGE, AISI-ST/SC-RGD×1, AISI-ST/SC-RGD×2, and AISI-ST/SC-RGD×4, were co-incubated with 4T1 or H22 tumor cells for 2 h (MOI 100). The free bacteria were removed, and adherent tumor cell strains were detected by comparing the total fluorescence intensity. Chassis-engineered strains stably expressing red fluorescent protein (RFP) were used. *n*=5. Data are presented as means±SD. $^{*}P<0.05$, $^{***}P<0.001$, $^{****}P<0.0001$, ns non-significant. AISI. Attenuated *Salmonella* Δ*htrA*::*luxI*-VNP20009 strain; ST. SpyTag; SC. SpyCatcherΔ; RGD. Arginine-glycine-aspartic acid; RGE. Arginine-glycine-glutamic acid; BSA. Bovine serum albumin; eGFP. Enhanced green fluorescent protein; Lys. L-lysine; Asp. Aspartic acid

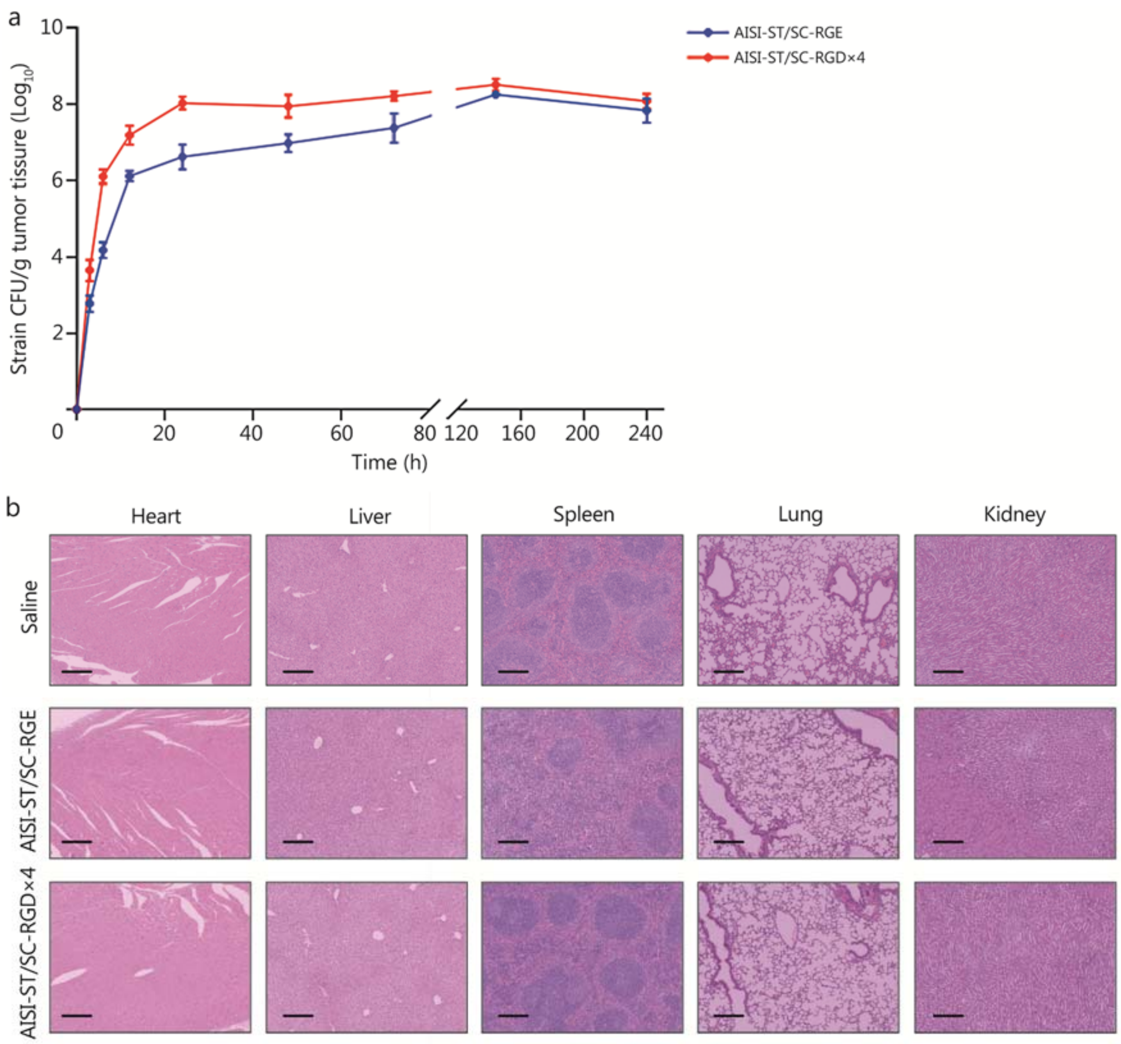

Fig. S4 The AISI-ST/SC-RGD×4 strain display excellent tumor-targeting properties and biosafety. **a** Time-dependent changes in bacterial titers within tumors for the AISI-ST/SC-RGE and AISI-ST/SC-RGD×4 strains in H22 cell subcutaneous xenograft model mice. n=4. **b** Representative H&E images of individual organs 12 h after the administration of the saline, AISI-ST/SC-RGE or AISI-ST/SC-RGD×4 strains to H22 cell subcutaneous xenograft model mice. Scale bars=40 μm. Data are presented as means±SD. $^{*}P$<0.05, $^{***}P$<0.001, $^{****}P$<0.0001. AISI. Attenuated *Salmonella* Δ*htrA*::*luxI*-VNP20009 strain; ST. SpyTag; SC. SpyCatcherΔ; RGD. Arginine-glycine-aspartic acid; RGE. Arginine-glycine-glutamic acid

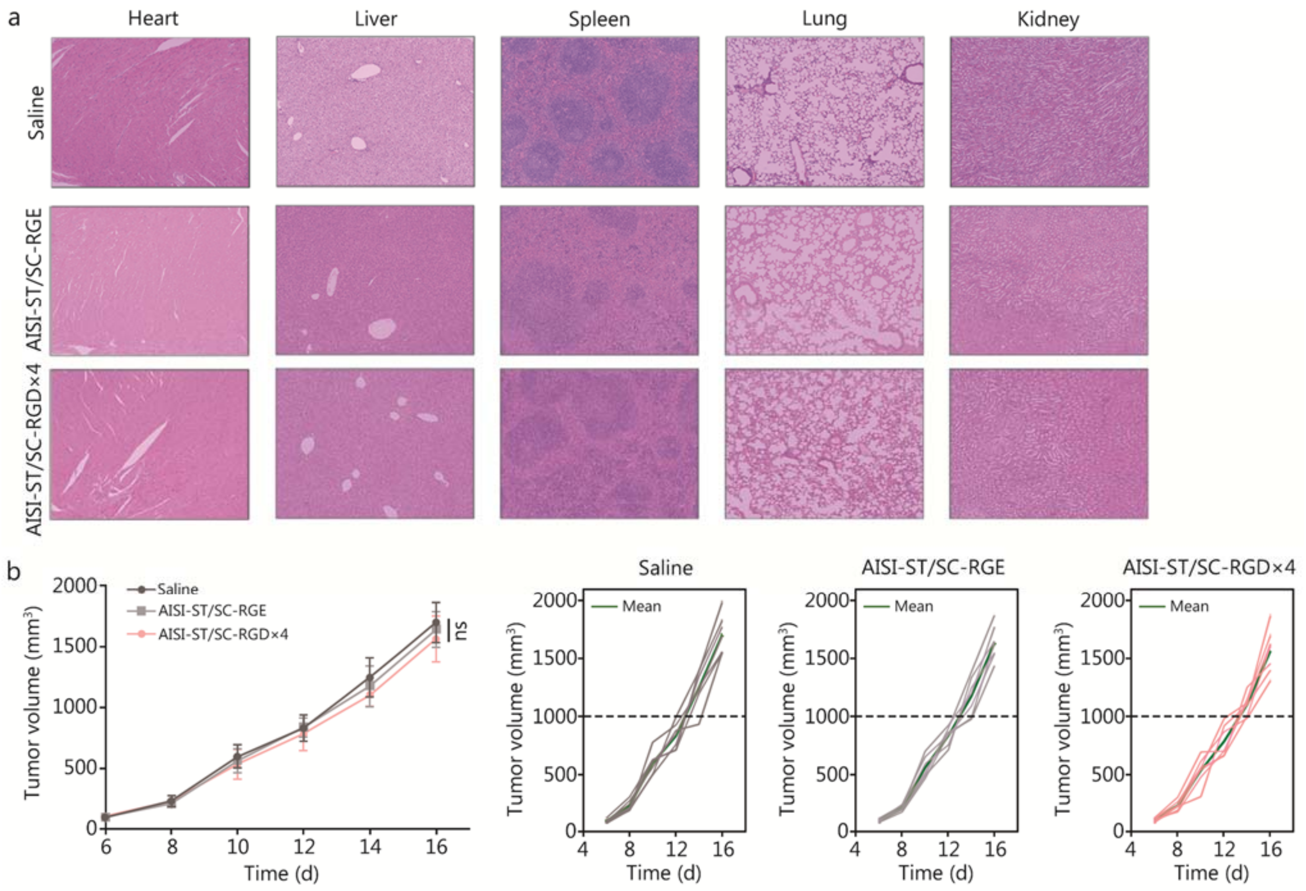

Fig. S5 The AISI-ST/SC-RGD×4 strain exhibits no chronic toxicity but have weak anticancer effects. **a** Representative H&E images of individual organs 10 d after treatment of B16-F10 tumor-bearing mice with different engineered strains. Scale bars=40 μm. **b** Comparison of anticancer effects of different engineered strains, including AISI-ST/SC-RGE and AISI-ST/SC-RGD×4, with saline-only administration as a control (left). The individual tumor growth variation curves are shown (right). *n*=7. Data are presented as means±SD. ns. Non-significant; AISI. Attenuated *Salmonella* Δ*htrA*::*luxI*-VNP20009 strain; ST. SpyTag; SC. SpyCatcherΔ; RGD. Arginine-glycine-aspartic acid; RGE. Arginine-glycine-glutamic acid

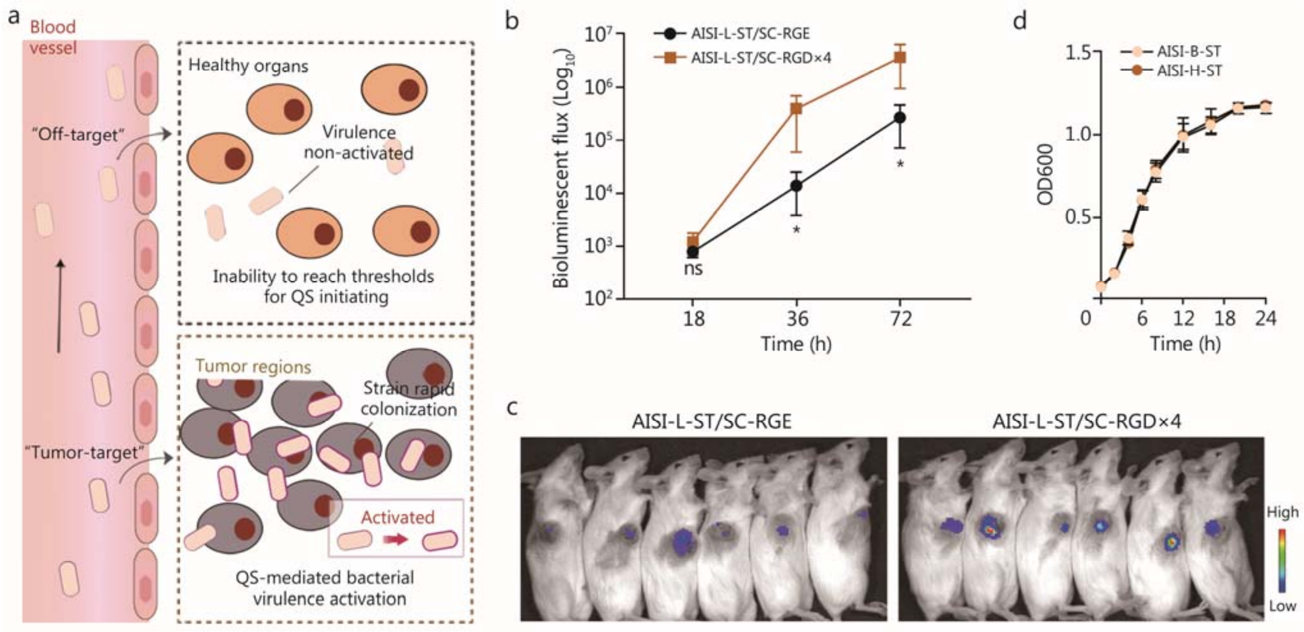

Fig. S6 The AISI-ST/SC-RGD×4 strain was coupled to the quorum-sensing (QS) system to achieve rapid tumor-specific initiation of target protein expression. **a** Schematic diagram of the AISI-ST/SC-RGD×4 strain coupled with a QS system to achieve tumor-specific expression of HtrA. The AISI-ST/SC-RGD×4 strain is highly enriched and proliferates specifically within the tumor, with the QS system initiating the expression of the HtrA protein. **b** Changes in bacterial density and bioluminescence flux in tumors 18, 36, and 72 h after administration. *n*=6. AISI-L-ST/SC-RGE strain: AISI-ST/SC-RGE strain carrying the pLuxI-LuxCDABE plasmid; AISI-L-ST/SC-RGD×4 strain: AISI-ST/SC- RGD×4 strain carrying the pLuxI-LuxCDABE plasmid. **c** Representative animal images from (b) at hour 36. **d** Comparison of the growth curves of the AISI-B-ST strain and the AISI-H-ST strain. *n*=3. Data are presented as means±SDs. $^{*}P<0.05$, ns non-significant. AISI. Attenuated *Salmonella* Δ*htrA*::*luxI*-VNP20009 strain; ST. SpyTag; SC. SpyCatcherΔ; RGD. Arginine-glycine-aspartic acid; RGE. Arginine-glycine-glutamic acid; L. LuxCDABE

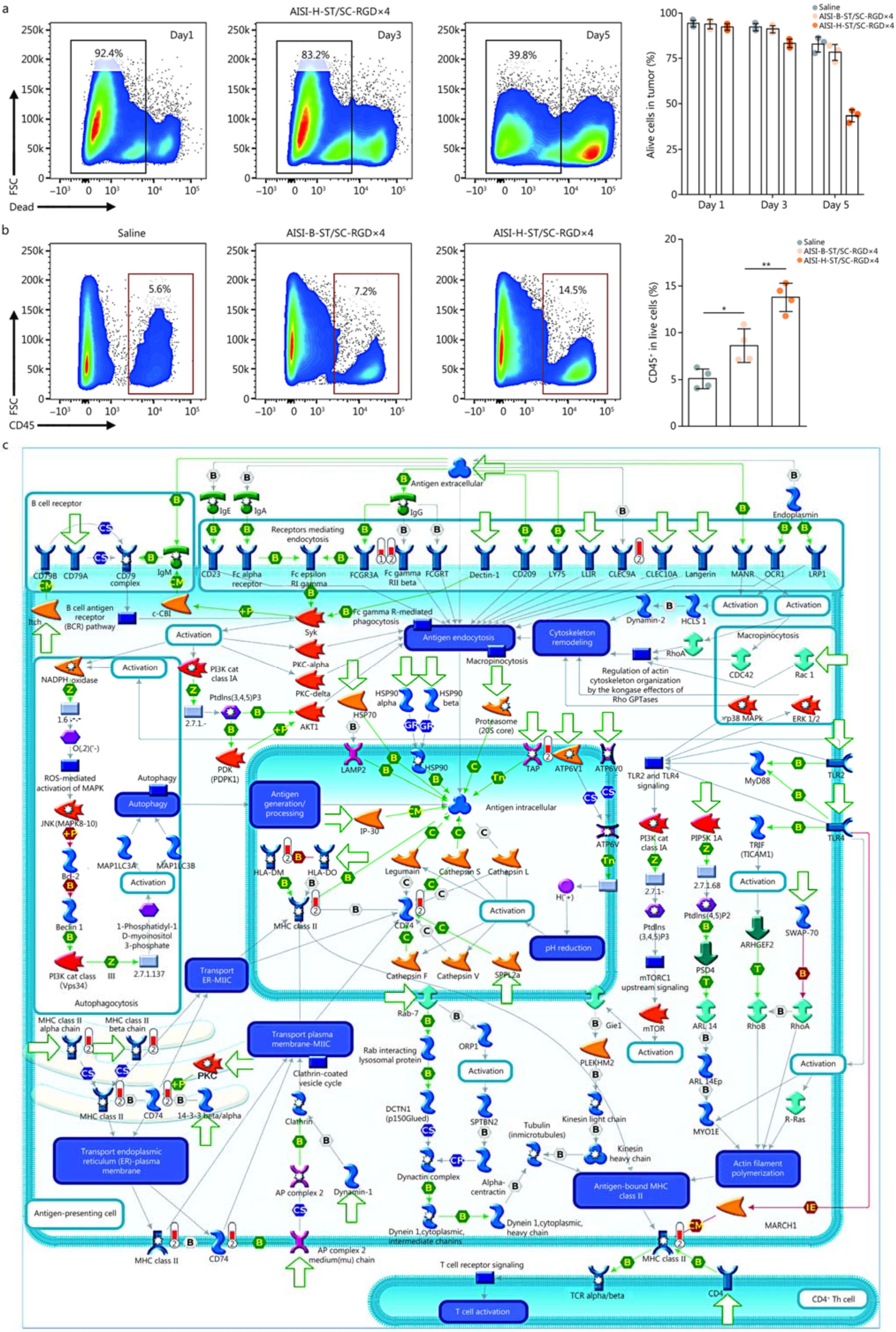

a
AISI-H-ST/SC-RGD×4
Day1
Day3
Day5
92.4%
83.2%
39.8%
FSC
Dead
Saline
AISI-B-ST/SC-RGD×4
AISI-H-ST/SC-RGD×4
Alive cells in tumor (%)
Day 1
Day 3
Day 5
b
Saline
AISI-B-ST/SC-RGD×4
AISI-H-ST/SC-RGD×4
5.6%
7.2%
14.5%
CD45
CD45⁺ in live cells (%)
c
Antigen extracellular
B cell receptor
Receptors mediating endocytosis
CD79B
CD79A
CD79 complex
IgM
IgE
IgA
IgG
CD23
Fc alpha receptor
Fc epsilon RI gamma
FCGR3A
Fc gamma RII beta
FCGRT
Dectin-1
CD209
LY75
LLIR
CLEC9A
CLEC10A
Langerin
MANR
OCR1
LRP1
Endoplasmin
Itch
B cell antigen receptor (BCR) pathway
c-CBl
Syk
Fc gamma R-mediated phagocytosis
Antigen endocytosis
Cytoskeleton remodeling
Dynamin-2
HCLS 1
Activation
Macropinocytosis
RhoA
CDC42
Rac 1
Regulation of actin cytoskeleton organization by the kongase effectors of Rho GPTases
p38 MAPk
ERK 1/2
NADPH oxidase
PI3K cat class IA
PKC-alpha
PKC-delta
HSP70
HSP90 alpha
HSP90 beta
Proteasome (20S core)
PtdIns(3,4,5)P3
AKT1
PDK (PDPK1)
LAMP2
HSP90
TAP
ATP6V1
ATP6V0
TLR2 and TLR4 signaling
MyD88
TLR2
TLR4
ROS-mediated activation of MAPK
Autophagy
JNK(MAPK8-10)
Bcl-2
Beclin 1
PI3K cat class III (Vps34)
1-Phosphatidyl-1 D-myoinositol 3-phosphate
MAP1LC3A
MAP1LC3B
Autophagocytosis
Antigen generation/ processing
IP-30
Antigen intracellular
ATP6V
HLA-DM
HLA-DO
MHC class II
CD74
Legumain
Cathepsin S
Cathepsin L
Cathepsin F
Cathepsin V
SPPL2a
H(+)
pH reduction
PI3K cat class IA
PIP5K 1A
TRIF (TICAM1)
PtdIns(4,5)P2
PtdIns (3,4,5)P3
mTORC1 upstream signaling
ARHGEF2
SWAP-70
PSD4
Transport ER-MIIC
Transport plasma membrane-MIIC
Rab-7
Rab interacting lysosomal protein
ORP1
Gie1
mTOR
PLEKHM2
ARL 14
ARL 14Ep
RhoB
MYO1E
R-Ras
MHC class II alpha chain
MHC class II beta chain
PKC
14-3-3 beta/alpha
Clathrin-coated vesicle cycle
Clathrin
DCTN1 (p150Glued)
SPTBN2
Tubulin (inmicrotubules)
Kinesin light chain
Kinesin heavy chain
Actin filament polymerization
Transport endoplasmic reticulum (ER)-plasma membrane
AP complex 2
Dynamin-1
Dynactin complex
Alpha-centractin
Dynein 1,cytoplasmic, intermediate chains
Dynein 1,cytoplasmic, heavy chain
Antigen-bound MHC class II
MARCH1
Antigen-presenting cell
AP complex 2 medium(mu) chain
T cell receptor signaling
TCR alpha/beta
CD4
T cell activation
CD4⁺ Th cell

Fig. S7 Changes in the tumor microenvironment after treatment with different engineered bacteria. **a** Detection of live cells in the tumor on day 1, 3 and 5 by flow cytometry. Representative flow cytometry plots from the AISI-H-ST/SC-RGD×4 strain group, which exhibited the most pronounced changes, are shown (left). *n*=3. **b** Detection of changes in the percentage of immune cells in the tumor on day 3 after treatment. *n*=4. **c** Gene ontology pathway map analyses of genes whose expression was altered in either the AISI-B-ST/SC-RGD×4 group or the AISI-H-ST/SC-RGD×4 group compared with the saline group. The AISI-H-ST/SC-RGD×4 strain effectively activates the "Immune response&Antigen presentation" pathway in tumors. Thermometers indicate gene dysregulation (red: upregulated; blue: downregulated; thermometer levels correspond to the level of dysregulation). Thermometers with number 1 represent the changed gene in AISI-B-ST/SC-RGD×4 group vs. Saline group, and number 2 in AISI-H-ST/SC-RGD×4 group vs. Saline group. Interactions between objects: green (positive or activation); red (negative or inhibition); gray (unspecified). B-Binding (physical interaction between molecules), TR-transcription regulation (physical binding of a transcription factor to the target gene promoter), +p-phosphorylation (protein activity is altered via addition of a phosphate group). More logo annotations can be found in the instructions (https://portal.genego.com/legends/MetaCoreQuickReferenceGuide.pdf). The data are reported as the means±SDs. $^{*}P<0.05$, $^{**}P<0.01$. Abbreviation names for all genes in GO pathway map are provided in full on the following website: https://portal.genego.com/legends/MetaCoreQuickReferenceGuide.pdf. FSC. Forward scatte; AISI. Attenuated *Salmonella* Δ*htrA*::*luxI*-VNP20009 strain; ST. SpyTag; SC. SpyCatcherΔ; B. Blank; H. HtrA high-temperature requirement

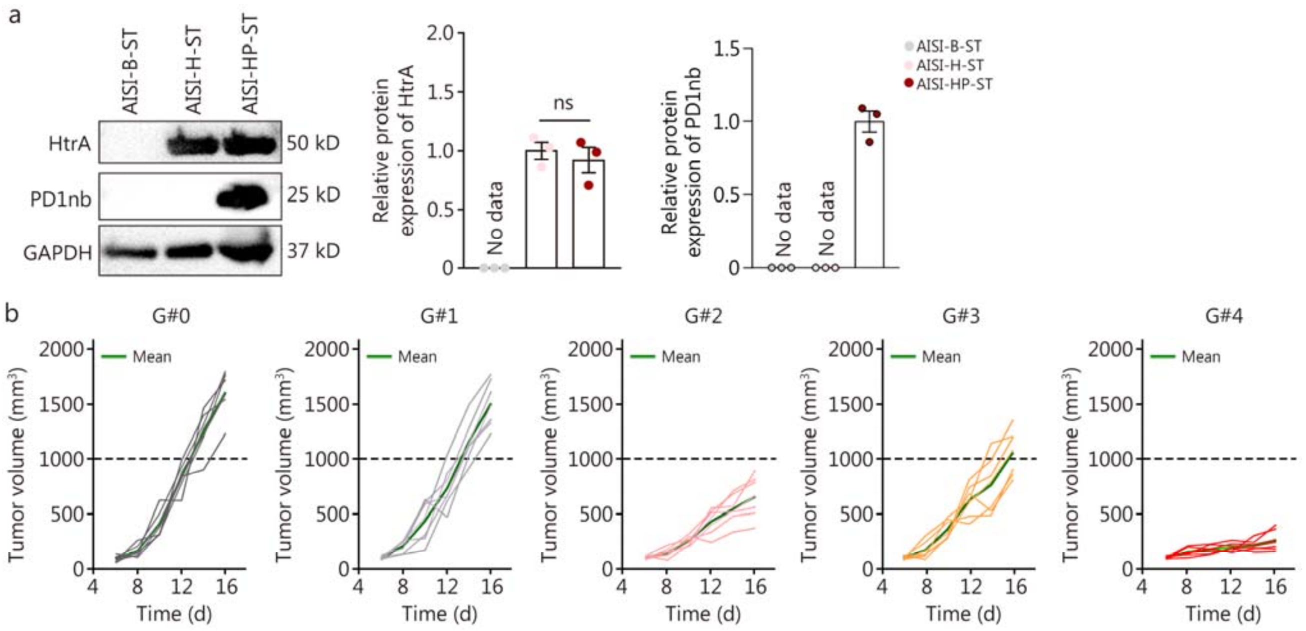

Fig. S8 The AISI-HP-ST/SC-RGD×4 strain demonstrates effective anticancer effects. **a** The protein expression of HtrA and PD1nb in different strains was evaluated via Western blotting (left). The protein expression in different strains were quantitatively analyzed (right). **b** Tumor growth curves for each mouse in the different groups in **Fig. 6i**, including AISI-B-ST/SC-RGD×4 (G#1 group), AISI-HtrA-ST/SC-RGD×4 (G#2 group), AISI-PD1nb-ST/SC-RGD×4 (G#3 group), and AISI-HtrA&PD1nb-ST/SC-RGD×4 (G#4 group). Saline (G#0 group) was used as the blank control. *n*=7. AISI. Attenuated *Salmonella* Δ*htrA*::*luxI*-VNP20009 strain; ST. SpyTag; B. Blank; H. HtrA high-temperature requirement A; PD1nb. Programmed cell death protein 1 nanobody; HP. HtrA and PD1nb; GAPDH. Glyceraldehyde-3-phosphate dehydrogenase

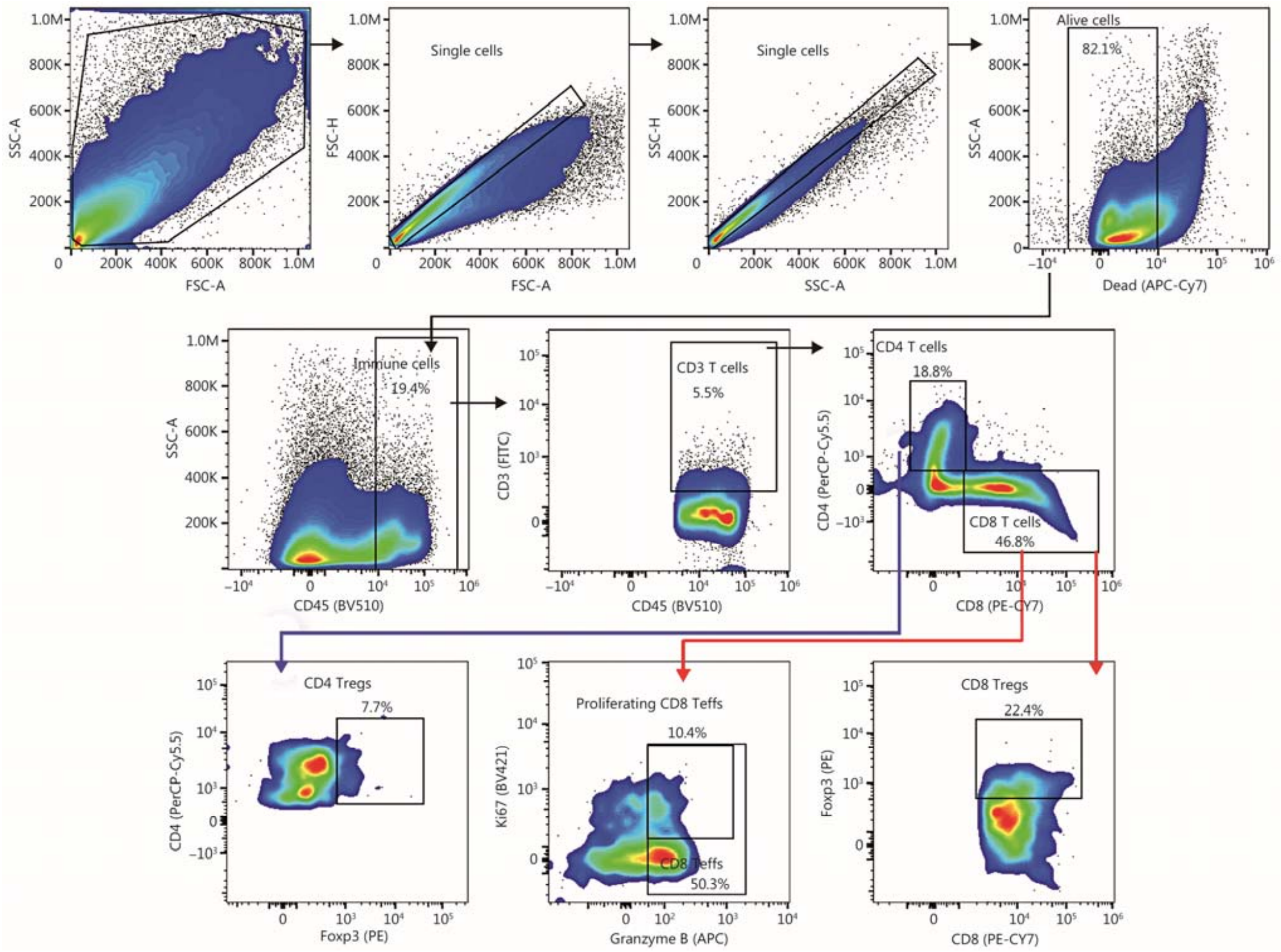

Fig. S9 Representative gating strategy for identifying lymphocytes with different phenotypes. FSC-A. Forward scatter area; SSC-A. Side scatter area; APC. Allophycocyanin; CD. Cluster of differentiation; BV. Brilliant violet; PE. Phycoerythrin; Cy. Cyanine; $CD4^+$ T cells. Cluster of differentiation 4-positive T lymphocyte; $CD8^+$ T cells. Cluster of differentiation 8-positive T lymphocyte; Teff. Effector T cells; Treg. Regulatory T cells
