# Supplementary material for: Octopus-inspired engineered bacteria with a plug-and-play surface display system achieves enhanced tumor-specific colonization and antitumor immunity
Source: Mil Med Res. 2026 Apr 27;13(1):100030. doi: 10.1016/j.mmr.2026.100030 (PMC13138155; doi:10.1016/j.mmr.2026.100030)
Supplement: Supplementary file 2 — Supplementary material [file mmc2.pdf]

Methods

Cells and strains

B16F10 mouse melanoma cells, 4T1 mouse breast carcinoma cells, H22 mouse hepatoma cell lines, and RAW264.7 mouse macrophage lines were preserved in our laboratory. All the cells were cultured in medium supplemented with 10% fetal bovine serum (FBS) and mycoplasma removal agents (40607ES03, Yeasen, Shanghai, China).

Plasmids harboring the J23100 promoter for target protein expression and the Axe/Txe (AT) system for plasmid stability were stored in the laboratory. Additionally, the pTD103luxI plasmid, containing the pLuxI promoter and a quorum-sensing positive feedback element, was obtained from Addgene (https://www.addgene.org/). Specific target genes were tagged at the C-terminus for detection. The main bacterial strains used in this study are described below. (1) An attenuated *Salmonella* Δ*htrA*::*luxI*-VNP20009 strain expressing OmpA-SpyTag (AISI-ST): the gene encoding OmpA-GS-ST-GS was inserted into the pTD103(Cm) plasmid backbone, which contains a p15A origin of replication (copy number: 10–15 per cell), a J23100 promoter driving expression of the target gene OmpA-GS-ST-GS, and a B0010 terminator preventing transcriptional readthrough. Chloramphenicol resistance (CmR) is employed as a selectable marker. (2) AISI-red fluorescent protein (RFP): the gene encoding RFP was inserted into the pTh23 plasmid backbone, which contains a pBR322 origin of replication (copy number: 15–20 per cell), a J23100 promoter driving expression of the target gene *rfp*, a B0010 terminator preventing transcriptional readthrough, and an Axe/Txe toxin-antitoxin system for preventing plasmid loss. Kanamycin resistance (KanR) is employed as a selectable marker. (3) AISI-pLuxI-LuxCDABE: the gene encoding LuxCDABE for quorum-sensing-regulated expression was inserted into the pTD103luxI plasmid backbone, which contains a pBR322 origin of replication (copy number: 500–700 per cell), a pLux promoter driving the expression of the target gene B0010 terminator to prevent transcriptional readthrough, and an Axe/Txe toxin-antitoxin system to prevent plasmid loss. Kanamycin resistance (KanR) was used for selection. (4) AISI-HtrA-ST (AISI-H-ST): the gene encoding HtrA for quorum-sensing-regulated expression was inserted into the pTD103luxI plasmid backbone and transformed into the AISI-ST strain. (5) AISI-PD1nb-ST (AISI-P-ST): the gene encoding PD1nb for quorum-sensing-regulated expression was inserted into the pTD103luxI plasmid backbone and transformed into the AISI-ST strain. (6) AISI-HtrA&PD1nb-ST (AISI-HP-ST): the genes encoding HtrA and PD1nb for quorum-sensing-regulated expression, which do not interfere with

each other, were inserted together into the pTD103luxI plasmid backbone and transformed into the AISI-ST strain. Representative plasmid maps are provided in **Additional file 2: Fig. S1.**

The DH5α and BL21 strains were obtained from Vazyme (Nanjing, China). All plasmids were constructed using the ClonExpress II/MultiS One-Step Cloning Kit (C112/C113, Vazyme, Nanjing, China). Plasmid assembly and amplification were conducted in DH5α *Escherichia coli* via heat shock-mediated transformation, followed by electroporation into either wild-type VNP20009 competent cells or genetically modified VNP20009 competent cells. Strains harboring empty plasmids were used as controls. For protein expression, plasmids were introduced into BL21 *Escherichia coli* via heat shock-mediated transformation. Representative plasmid maps are provided in **Additional file 2: Fig. S1**.

Prediction and visualization of protein-protein interactions

AlphaFold2 was employed using the methodology described by the DeepMind team to investigate the structure of SpyCatcherΔ (SC)-eGFP. AlphaFold2 was implemented in a CentOS 7 environment, with a comprehensive sequence database downloaded as a reference. The default deep learning parameters were used, enabling multiple sequence alignment (MSA) and template-based modeling to generate high-confidence protein structures. Model quality was assessed based on the predicted local distance difference test (pLDDT) scores and predicted aligned error (PAE) matrices. The final structural model was selected based on the highest mean pLDDT score and the lowest overall PAE score. For structural visualization, PyMOL was used to generate molecular representations. The protein backbones are displayed in cartoon mode, with the SC, arginine-glycine-aspartic acid (RGD), and enhanced green fluorescent protein (eGFP) domains highlighted in distinct colors. Additional structural details are provided in **Additional file 1: Fig. S2d**.

Bacterial real-time growth curve assays

The growth curves of different engineered attenuated *Salmonella* strains in Luria-Bertani (LB) medium were monitored via a microplate reader (Biotek, Winooski, USA). Briefly, 10 μl of VNP suspension (OD600=1.0) was inoculated into 1 ml of LB medium and distributed into 96-well plates at 200 μl per well. The plates were incubated at 37 °C for 20 h, and the OD600 was measured every 30 min via a brown optical filter at a wavelength of 600 nm to assess bacterial growth dynamics.

Protein expression and purification

A 6×His tag was added to the N-terminus of each protein sequence by cloning into the pET-28a

expression vector, facilitating Ni-affinity purification and detection. For protein expression, recombinant plasmids were transformed into BL21 (DE3) *Escherichia coli* via heat shock. The bacterial cultures were expanded at 37 °C until the OD600 reached 0.8, followed by induction with 1 mmol/L isopropyl β-D-1-thiogalactopyranoside (IPTG) at 16 °C and 180 rpm for protein expression.

Proteins were purified via Ni-affinity chromatography, and the purity of the eluted proteins was assessed via sodium dodecyl sulfate-polyacrylamide gel electrophoresis (SDS-PAGE). The target proteins were dialyzed in phosphate-buffered saline (PBS) buffer, and the protein concentration and purity were determined via SDS-PAGE and bicinchoninic acid (BCA) assay kits (Beyotime, P0012, Shanghai, China).

Quantification of SC-eGFP conjugated to AISI-ST strain

To quantify SC-eGFP conjugated to AISI-ST strain, a standard curve of fluorescence intensity versus protein mass was first established using serial dilutions of SC-eGFP (0, 5, 10, 15, 20 mg/ml) measured by a microplate reader at excitation/emission wavelengths of 488/507 nm. Subsequently, 3×10^8 CFU of AISI-ST strain was incubated with 3 mg of SC-eGFP (37 °C, 1 h), washed three times by centrifugation, and resuspended in PBS. The fluorescence intensity of the suspension was measured, and the bound protein mass was calculated using the standard curve. Based on this formul $N(SC\text{-}eGFP)=\frac{m(SC\text{-}eGFP)NA}{M(SC\text{-}eGFP)N(AISI\text{-}ST)}$, the amount of SC-eGFP bound to a single AISI-ST strain can be calculated. *N* is the number of the substance; *NA* is the Avogadro constant, approximately 6.02×10^{23} mol^{-1}; *m* is the mass of the substance; *M* is the molar mass.

Adhesion assay of engineered bacteria

To evaluate the ability of different engineered bacterial strains to adhere to tumor cells, 4T1 and B16F10 cells were seeded into 96-well plates and incubated overnight. Subsequently, 1×10^7 bacteria from different engineered strains were coincubated with 1×10^5 tumor cells for 2 h. After incubation, the supernatant was removed, and nonadherent or weakly adherent bacteria were washed away by gently rinsing 1–2 times with PBS. For H22 suspension cells, cells were seeded into 6-well plates and coincubated with engineered bacteria at a multiplicity of infection (MOI) of 100:1 at 37 °C. After a 2-hour static incubation, the supernatant was removed by centrifugation at 800 rpm for 3 min, followed by three PBS washes to eliminate nonadherent or weakly adherent bacterial strains. The adhesion of different bacterial strains to various tumor cells was visualized via fluorescence microscopy (Carl Zeiss,

Germany). For a more accurate quantification of bacterial adhesion to tumor cells, the fluorescence intensity was measured via a microplate reader (Biotek, Winooski, USA) at excitation/emission wavelengths of 532/588 nm.

Biofilm formation assays

To assess bacterial biofilm-mediated adhesion, activated bacterial strains were passaged at a 1:100 dilution into fresh culture medium and grown at 37 °C with shaking at 220 rpm until they reached an OD600 of 0.6–0.8. The cultures were then diluted 1:100 into fresh M63 medium in glass tubes and incubated statically at 37 °C with gentle shaking at 100 rpm for 36 h. After incubation, the tubes were washed twice with PBS, air-dried, and stained with 2% crystal violet. The biofilm appeared as a purple ring at the air-liquid interface. To quantify biofilm formation, the stained biofilm was dissolved in 95% ethanol, and the absorbance at 570 nm (OD_{570}) was measured.

Electron microscopy imaging

For the scanning electron microscopy (SEM) analysis, bacterial samples were prepared using previously described protocols. Briefly, AISI-SD and AISI-ST strains were cultured overnight in LB media supplemented with kanamycin at 37 °C. The activated bacterial strains were subsequently inoculated into 4–5 ml of fresh Luria-Bertani (LB) medium and grown to the stationary phase. Bacteria were then harvested by centrifugation at 8000 rpm for 3 min, and the resulting pellet was resuspended in 2.5% glutaraldehyde for fixation at room temperature in the dark for at least 30 min. Sample preparation and imaging were performed by Wuhan Saville Company.

For the transmission electron microscopy (TEM) analysis of extracellular polysaccharide (EPS), bacterial samples were collected, stained, and fixed using previously described protocols. Briefly, bacterial samples were kept on ice and processed in an osmotically balanced buffer solution containing 2.5% glutaraldehyde, 2% paraformaldehyde, 0.1 mol/L sodium cacodylate, 0.9 mol/L sucrose, 10 mmol/L $CaCl_2$, 10 mmol/L $MgCl_2$, 0.075% ruthenium red, and 75 mmol/L lysine acetate. The samples were rinsed three times with a 0.075% ruthenium red buffer solution and subsequently transferred to fixation buffer containing 0.075% ruthenium red and 1% osmium tetroxide on ice for 1–2 h. For dehydration, the samples were sequentially treated with gradients of different solutions: 30%, 50%, 70%, and 80% ethanol (15 min each); 90% and 95% acetone (15 min each); and 100% anhydrous acetone (20 min each). Next, the samples were incubated at room temperature in a 1:1 mixture of anhydrous acetone and Spurr resin for 1 h, immersed in a 1:3 acetone-to-Spurr resin mixture for 3 h,

and then left overnight. The samples were subsequently embedded in Spurr resin in Eppendorf tubes and polymerized at 70 °C for at least 9 h. Ultrathin sections were prepared using a Leica EM UC7 ultramicrotome, stained with uranyl acetate and lead citrate for 5–10 min, and imaged with a Hitachi HT-7700 transmission electron microscope.

Animal experiments

B16F10 melanoma cells (2×10^5 cells per mouse) were subcutaneously injected into the right axillary region of C57BL/6 mice. H22 hepatoma cells (1×10^6 cells per mouse) were subcutaneously injected into the right axillary region of BALB/c mice. 4T1 breast cancer cells (5×10^5 cells per mouse) were subcutaneously injected into the right mammary fat pads of BALB/c mice. For tumor efficacy evaluation experiments, each group shall contain at least 7 mice. The specific numbers of mice for other related animal experiments can be found in the figure legends. When the tumors reached a volume of 80–120 mm^3, different engineered bacterial strains (2×10^6 CFU per mouse) were administered via a tail vein injection. The tumor dimensions were measured using Vernier calipers every 1–2 d, and the tumor volume (V, mm^3) was calculated with the formula $V=a^2b\times0.52$, where “a” represents the smaller diameter and “b” represents the larger diameter. Tumor growth curves were plotted to track tumor progression over time for individual mice in each experimental group. Additionally, body weight changes were monitored throughout the study. The mice were monitored daily for signs of distress, tumor necrosis, or other adverse reactions to assess survival rates. Humane endpoints were defined as a tumor weight reaching 10% of the total body weight, at which point euthanasia was performed, and the event was recorded as death.

4T1 cells (5×10^5 cells per mouse) were injected into the tail vein of 6-week-old female BALB/c mice to establish a 4T1 lung metastasis model. After approximately 5 d, when initial lung metastases had formed, different engineered bacterial strains (2×10^6 CFU per mouse) were administered via a tail vein injection. The control group received an equivalent volume of saline instead of the bacterial strains. At designated time points, lung tissues were collected, photographed, weighed, and analyzed for the metastatic burden.

Bacterial biodistribution

Various engineered bacterial strains were injected into mice bearing B16-F10 melanoma, 4T1 breast tumors, or H22 subcutaneous tumors via the tail vein. At different time points after administration (12

h for solid tumors and 6 h for lung metastases), the mice were euthanized, and their tumors, hearts, livers, spleens, lungs, and kidneys were harvested. These tissues were cut into pieces and placed in PBS solution containing 0.5% Triton X-100. A tissue homogenizer (70 Hz, 60 s per cycle, 10 s interval, 3 cycles total) was used to achieve complete homogenization and lysis. Then, the supernatant obtained from the homogenization was diluted and uniformly spread onto the corresponding resistant agar plates. The agar plates were cultured at 37 °C overnight. The bacterial colonies were counted, and the bacterial titer per gram of tissue was calculated. The bacterial titer in the tumors was compared with that in different organs to evaluate the tumor-targeting ability of different strains.

In addition, engineered bacterial strains carrying the LuxCDABE plasmid [2×10^6 colony-forming units (CFU) per mouse] were injected into tumor-bearing mice via the tail vein. At different time points, an *in vivo* imaging system (IVIS Lumina III, PerkinElmer, Waltham, MA, USA) was used to monitor the fluorescence signal of LuxCDABE to characterize the colonization of these strains at the tumor site. For enhanced visualization of bacterial localization in different organs, the mice were euthanized, and the organs were collected and subjected to real-time *ex vivo* imaging postadministration.

Tumor tissue RNA-seq

B16-F10 cells were inoculated into C57BL/6 mice (7 weeks old, female) in the right axillary region. The mice were intraperitoneally injected with saline or different strains (2.0×10^6 CFU per mouse) after tumor formation. Three days later, the tumor tissues were collected. To avoid spatial heterogeneity, the entire tumor was homogenized and thoroughly mixed before RNA extraction from the tissue. The extracted RNA was then subjected to RNA sequencing, which was conducted by Shanghai Oebiotech. For the analysis of differentially expressed genes (DEGs) and functional enrichment, the Limma R package was used to obtain $\log_2$ FC and *P*-adjust values between groups. Genes with $|\log_2 \text{FC}|>1$ and *P*-adjust<0.05 were considered to be significantly differentially expressed. For functional enrichment analysis, the ClusterProfiler R package was used to perform Gene Ontology (GO) enrichment analysis of the genes significantly upregulated between groups. The expression of key pathway genes from the enrichment results was visualized via a heatmap. The complete transcriptomic data have been uploaded to the Gene Expression Omnibus (GSE250146). The significant lists were uploaded from a Microsoft Excel spreadsheet to MetaCore for pathway analysis (Clarivate Analytics).

Flow cytometry

After the tumor-bearing mice were euthanized, the tumors were excised, minced, and cultured in

digestion medium [containing 10 U/ml collagenase I, 400 U/ml collagenase IV, and 30 U/ml DNase I in Hank's balanced salt solution (HBSS)] for 30 min. The resulting mixture was passed through a 40 μm cell filter to eliminate cell clumps, yielding a single-cell suspension. An erythrocyte lysis solution (R1010, Solarbio, China) was added, and the suspension was kept on ice for 1–2 min to lyse red blood cells. The cells were washed with PBS and stained with a fixable viability dye (564407, BD, USA) for 15–20 min in the dark at room temperature. Following this, staining with mouse-specific antibodies was performed as outlined in **Additional file 2: Table S1**, and the cells were fixed with a membrane-fixing solution (565388, BD, USA). To assess the bacterial load, the weight of each organ was recorded, and 0.5% Triton X-100 was added according to the tissue weight (2 μl/mg). The tissues were homogenized, stored at 4 °C for 30 min, and then passed through a cell strainer to remove clumps. Bacteria with RFP fluorescence in the lysate were identified via flow cytometry. Strain-induced apoptosis was detected according to previously published protocols. All analyses were conducted via a BD Canto II flow cytometer.

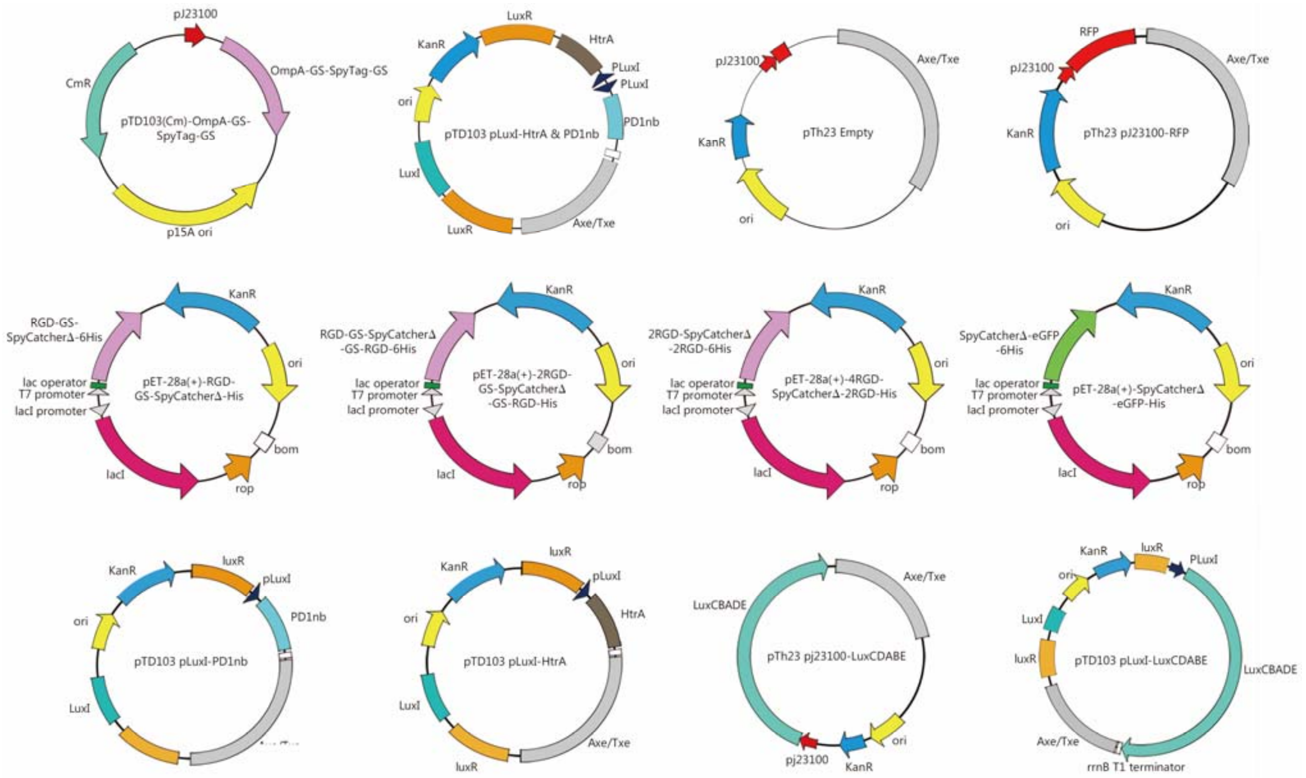

Fig. S1 Representative plasmid profiles used in this study. OmpA. Outer membrane protein A; HtrA. High-temperature requirement A; PD1nb. Programmed cell death protein 1 nanobody; RFP. Red fluorescent protein; eGFP. Enhanced green fluorescent protein; RFP. Red fluorescent protein; RGD. Arginine-glycine-aspartic acid

Table S1 List of antibodies used for flow cytometry analyses in this study

Antibody	Fluorescence	Supplier	Catalog No.
CD45	PE-Cy7	BD Biosciences, Franklin Lakes, USA	552848
CD45	BV510	BD Biosciences, Franklin Lakes, USA	740131
CD3	FITC	BD Biosciences, Franklin Lakes, USA	561798
CD11b	FITC	BD Biosciences, Franklin Lakes, USA	561688
CD11b	APC	BD Biosciences, Franklin Lakes, USA	561690
F4/80	APC	BD Biosciences, Franklin Lakes, USA	566787
CD11c	PE-Cy7	BD Biosciences, Franklin Lakes, USA	561022
CD80	BV421	BD Biosciences, Franklin Lakes, USA	566285
Ki67	BV421	BD Biosciences, Franklin Lakes, USA	562899
MHCII	FITC	BD Biosciences, Franklin Lakes, USA	562009
CD4	PerCP-Cy5.5	BD Biosciences, Franklin Lakes, USA	561115
CD8	PE-Cy7	BD Biosciences, Franklin Lakes, USA	561097
Foxp3	PE	BD Biosciences, Franklin Lakes, USA	560414
CD206	PE	Invitrogen, Carlsbad, USA	12-2061-82
CD206	APC	Invitrogen, Carlsbad, USA	17-2061-82
GzmB	APC	Invitrogen, Carlsbad, USA	17-8898-82
CD86	PE	Invitrogen, Carlsbad, USA	12-0862-82

CD. Cluster of differentiation; MHCII. Major histocompatibility complex class II; Foxp3. Forkhead box p3; GzmB. Granzyme B; PE. Phycoerythrin; BV. Brilliant violet; FITC. Fluorescein isothiocyanate; APC. Allophycocyanin; Cy7. Cyanine 7
